# Invention and characterization of a systemically administered, attenuated and killed bacteria-based multiple immune receptor agonist for anti-tumor immunotherapy

**DOI:** 10.3389/fimmu.2024.1462221

**Published:** 2024-11-07

**Authors:** Michael J. Newman

**Affiliations:** Indaptus Therapeutics, Inc., New York, NY, United States

**Keywords:** toll-like receptor, TLR agonist, bacteria, immunotherapy, innate, adaptive, anti-tumor, anti-cancer

## Abstract

Activation of immune receptors, such as Toll-like (TLR), NOD-like (NLR) and Stimulator of Interferon Genes (STING) is critical for efficient innate and adaptive immunity. Gram-negative bacteria (G-NB) contain multiple TLR, NOD and STING agonists. Potential utility of G-NB for cancer immunotherapy is supported by observations of tumor regression in the setting of infection and Coley’s Toxins. Coley reported that intravenous (i.v.) administration was likely most effective but produced uncontrollable toxicity. The discovery of TLRs and their agonists, particularly the potent TLR4 agonist lipopolysaccharide (LPS)-endotoxin, comprising ~75% of the outer membrane of G-NB, suggests that LPS may be both a critical active ingredient and responsible for dose-limiting i.v. toxicity of G-NB. This communication reports the production of killed, stabilized, intact bacteria products from non-pathogenic G-NB with ~96% reduction of LPS-endotoxin activity. One resulting product candidate, Decoy10, was resistant to standard methods of cell disruption and contained TLR2,4,8,9, NOD2 and STING agonist activity. Decoy10 also exhibited reduced i.v. toxicity in mice and rabbits, and a largely uncompromised ability to induce cytokine and chemokine secretion by human immune cells *in vitro*, all relative to unprocessed, parental bacterial cells. Decoy10 and a closely related product, Decoy20, produced single agent anti-tumor activity or combination-mediated durable regression of established subcutaneous, metastatic or orthotopic colorectal, hepatocellular (HCC), pancreatic, and non-Hodgkin’s lymphoma (NHL) tumors in mice, with induction of both innate and adaptive immunological memory (syngeneic and human tumor xenograft models). Decoy bacteria combination-mediated regressions were observed with a low-dose, oral non-steroidal anti-inflammatory drug (NSAID), anti-PD-1 checkpoint therapy, low-dose cyclophosphamide (LDC), and/or a targeted antibody (rituximab). Efficient tumor eradication was associated with plasma expression of 15-23 cytokines and chemokines, broad induction of cytokine, chemokine, innate and adaptive immune pathway genes in tumors, cold to hot tumor inflammation signature transition, and required NK, CD4+ and CD8+ T cells, collectively demonstrating a role for both innate and adaptive immune activation in the anti-tumor immune response.

## 1 Introduction

Immune checkpoint therapy has revolutionized the treatment of advanced or metastatic solid tumors, producing durable clinical responses in several indications (1). Despite this success, most patients do not respond, and some patients develop resistance to therapy after initial response, highlighting the continuing need for new or improved immunotherapies (2). Current approved immune checkpoint therapy is based on releasing tumor-mediated impediments to adaptive immune cell activation. Alternative approaches include enhancement of innate or adaptive immune cell migration, proliferation, maturation, activation, and antigen processing/presentation, all of which are facilitated to a significant extent by secreted cytokines and chemokines (3). Despite being principal inducers of positive innate and adaptive immune responses, expression of the same cytokines and chemokines at inappropriate times, places, levels and/or duration can contribute to the process of tumorigenesis, immunosuppression in the tumor environment, and systemic toxicity, with the latter two being of greatest concern in the setting of newly diagnosed advanced disease (4, 5).

Two cytokines are approved for cancer immunotherapy (IL-2 and interferon-alpha), but success with single cytokine approaches has been limited by both toxicity and limited efficacy (6). This may reflect a requirement for multiple different cytokines and chemokines for efficacy, resulting in dose-escalation to toxicity when single cytokines are used as monotherapies. In addition, a systemic response is required because tumors suppress systemic immunity and most of the steps involved in innate and adaptive immune cell mobilization, migration, proliferation, maturation, activation, and antigen processing/presentation take place outside of a tumor (7, 8). Finally, activation of both innate and adaptive pathways may be required for highly efficient or durable anti-tumor immune responses (9, 10). Thus, a key challenge for modern cancer immunotherapy is how to activate systemic innate and adaptive responses, for example via administration or induction of multiple cytokines and chemokines, without enhancing immune suppression in the tumor or eliciting systemic toxicity. A long-standing observation may provide a clue for one solution to this challenge.

In addition to immune surveillance of cancer, our immune systems evolved to eliminate pathogens by responding to common pathogen constituents or pathogen-associated molecular patterns (PAMPs) producing innate and adaptive immune pathway activation, mediated in part via induction of secretion of a broad array of cytokines and chemokines (11–13). Most of the steps required for innate and adaptive cellular immune responses are antigen and target cell non-specific, and specific steps involving antigen processing and presentation are enhanced non-specifically by cytokines and chemokines (14). Therefore, the long-standing observation of spontaneous tumor regression in the setting of bacterial infection is not surprising (15, 16). This observation was the basis for the world’s first immunotherapy, Coley’s Toxins, invented between 1891 and 1894, which was a heat-killed mixture of intact Gram-negative and Gram-positive pathogenic bacteria (17–19).

Coley’s Toxins was reported to produce durable responses in advanced cancer patients (20). The mechanism of action was not understood, and approximately fifteen different methods of manufacturing were reported, likely leading to significant variability in activity. In addition, Coley believed that his product worked best when it was administered intravenously, but it produced uncontrollable toxicity via this route, so most administration was intratumoral or intradermal (21, 22). This may also have contributed to variability in anti-tumor response, due to reduced ability to induce a systemic immune response. The US FDA decided not to grandfather in Coley’s Toxins as a prescription drug in 1963, and this, in conjunction with the advent of chemotherapy and radiotherapy, may have contributed to loss of interest in this approach.

The discovery of Toll-like (TLR), Nucleotide oligomerization domain (NOD)-like, and Stimulator of interferon genes (STING) receptor pathways and the presence of TLR, NOD and STING agonists in bacteria has provided information on both the mechanism of action of Coley’s Toxins and a likely source of its dose-limiting i.v. toxicity. Modern research supports a mechanism involving activation of immune cells by bacteria-associated PAMPs, including both direct activation, and indirect activation via induction of the secretion of multiple cytokines and chemokines (23–28).

The Gram-negative component of Coley’s Toxins was found to be the active bacterial component (17). Since lipopolysaccharide (LPS)-endotoxin represents approximately 75% of the outer membrane of Gram-negative bacteria and is a major causative agent of sepsis, it was probably responsible for the dose-limiting i.v. toxicity of Coley’s Toxins (29). High levels of LPS-endotoxin activity may also limit the amount of other immune activators, including other TLR, NOD and STING agonists, which can be administered systemically with wild-type bacteria, and which might be important synergy partners for effective immune responses. Despite the role of LPS in sepsis, activation of its receptor (TLR4) plays an important role in both innate and adaptive therapeutic immune responses, suggesting that LPS may have been both a source of dose limiting i.v. toxicity, as well as a potential major active ingredient in Coley’s Toxins (30–35). Purified LPS has been administered i.v. in cancer trials, producing very limited activity, suggesting that it was probably not the only constituent of Coley’s Toxins required for anti-tumor activity (36–40). Purified LPS has also been administered i.v. to over 1,000 healthy human volunteers and is generally well-tolerated at doses up to 4 ng/kg (41).

TLRs have been shown to be present in and to contribute directly to anti-tumor activities of essentially all major innate and adaptive cell types (42–48). TLR agonists have been approved as vaccine adjuvants for the prevention of cancer and viral diseases, and for treatment of superficial and non-metastatic tumors. One live bacterial product with TLR agonist activity, Bacillus Calmette-Guérin (BCG), is approved for non-muscle invasive bladder cancer (49, 50). All of the approved products, except mifamurtide for non-metastatic osteosarcoma, are administered locally. There are no TLR agonist or bacteria-related products approved for advanced or metastatic cancers, despite the exceptionally well-validated role of TLRs in activation of both innate and adaptive immune responses.

Systemically and locally administered single, untargeted and targeted TLR agonists have been shown to produce significant pre-clinical anti-tumor activity, including synergy with each other, local radiotherapy, chemotherapy, and checkpoint therapies (51–59). Mono-specific, unconjugated and antibody-conjugated, locally and systemically administered TLR agonists are currently being evaluated in clinical trials (26, 60).

Over the past 30 years, most intact bacteria-based cancer immunotherapy approaches have utilized live, attenuated bacteria, designed to selectively replicate in the tumor microenvironment and infect tumor cells. These studies have largely utilized either Gram-positive bacteria, lacking LPS-endotoxin, or Gram-negative bacteria engineered to eliminate LPS-endotoxin activity (61, 62). On the other hand, the original results with Coley’s toxins, a possible requirement for systemic immune activation, and the established role of TLR4 in bridging innate and adaptive immune responses support a systemic approach with killed, intact Gram-negative bacteria that retain reduced, but still substantial, levels of LPS-endotoxin activity.

Therefore, I have developed a process for reducing the level of LPS-endotoxin activity by ~96%, as well as killing and stabilizing single strains of non-pathogenic, Gram-negative bacteria. This communication describes the properties and anti-tumor activity of two related product candidates, Decoy10 and Decoy20. Based on a significant reduction in LPS-endotoxin activity, and the well-established rapid processing and clearance of intravenously administered bacteria by immune cells in the liver and spleen (63–65), my hypothesis is that Decoy product candidates may represent a multi-immune receptor agonist package that can be administered safely intravenously, producing anti-tumor activity via passively-targeted, pulse-priming of both innate and adaptive cellular immune pathways.

## 2 Materials and methods

All research was conducted at contract research organizations (CROs).

### 2.1 Compound manufacturing

Decoy10 was produced from *Escherichia* (*E.*) *coli* (Migula) Castellani and Chalmers (ATCC 13070), obtained from ATCC. Decoy20 was produced from *E. coli* Genetic Stock Center (CGSC) Strain 4558/AT984, obtained from the CGSC (New Haven, CT). Both strains are non-pathogenic K-12 derivatives, Biosafety Level 1 (BSL-1) and diaminopimelic acid (DAP) auxotrophs. Mammals do not produce DAP, preventing proliferation of live cells *in vivo*, and providing a failsafe mechanism in the event of incomplete cell killing. DAP-dependence also provides a method for strain confirmation during manufacturing (61).

Decoy10 and Decoy20 were manufactured by the same process at Molecular Diagnostic Services (MDS) (San Diego, CA). All phosphate-buffered saline (PBS) used for manufacturing and biological assays was calcium and magnesium-free. Bacteria were grown at 37°C on agar plates or in liquid culture with shaking using LB/Miller broth (Lennox LB Broth Base, Gibco #12780029, adjusted to 10 g/L NaCl), supplemented with 0.5% glucose, 1 mM DAP (Sigma #D1377-56) and 2 mM MgCl2 (growth medium). A single colony from a plate was grown overnight in 75 mL growth medium. Twelve mL of overnight culture was added to one liter of fresh growth medium in a 2.8-liter flask and incubated at 37°C with shaking (2-4 flasks per batch). Late log phase cells (O.D.600 of 1 = 1.12x10^9^ cells/mL) were harvested by washing twice with 4°C LB/Miller broth, 0.1 mM DAP, 20 mM MgCl2 by centrifugation at 2,000 x *g* at 4°C for 30 minutes. Washed cells were resuspended at 1x10^10^ cells/mL in the same medium at 4°C.

LPS-endotoxin activity was reduced by ≥90% by treating cells with 1 mg/mL polymyxin B (PMB, Calbiochem #5291) for 1 hour at 4°C, with gentle stirring. Cells were washed three times by centrifugation at 3,000 x *g* with 4°C PBS, pH 7.5, 20 mM MgCl2 (incubation medium), resuspended at 1x10^11^ cells/mL in the same 4°C incubation medium and then killed and stabilized by diluting 10-fold into 4°C incubation medium with 1% glutaraldehyde (GA, Sigma #G7651). The suspension was then incubated for 1 hour with gentle stirring at 4°C. The cells were washed three times by centrifugation with incubation medium (no GA) at 4°C, resuspended in 4°C 50% PBS, pH 7.5, 1 mM MgCl2, 12% trehalose (Acros #309840250) (freezing medium) at 1x10^11^ cells/mL, aliquoted into 2 mL cryovials (0.3 mL per vial), flash-frozen in liquid nitrogen and stored at -80°C. Samples were removed at each step and tested for cell concentration by O.D.600, viability by plating efficiency ± DAP, and LPS-endotoxin activity by Limulus Amebocyte Lysate (LAL) assay (Endosafe Endochrome K kinetic method, Charles River Laboratories [CRL]). Cellular dispersity and integrity were assessed by electron microscopy (EM) (University of California at San Diego EM facility) and/or optical microscopy after staining. GA-mediated cell stabilization was confirmed by optical microscopy after subjecting unprocessed parent bacteria and Decoy cell suspensions to sonication in a round bottom flask using a Fisher Scientific sonic dismembrator fixed with a microprobe at setting #3.

Dose-response and incubation time-course analyses were conducted with both PMB and GA in order to optimize LPS-endotoxin activity reduction and cell killing. Seven batches of Decoy10 and four batches of Decoy20 were produced with an average reduction of LPS-endotoxin activity of 96% (range of 92-99%). All seven batches of Decoy10 and three of the Decoy20 batches were used in the studies reported in this communication. The products appeared to be highly stable when stored frozen, based on consistency of LPS-endotoxin activity, and various *in vitro* and *in vivo* results obtained during storage of Decoy bacteria for several years.

### 2.2 *In vitro* studies

Human TLR, NOD, Dectin, Mincle, retinoic acid-inducible gene I (RIG-I), and melanoma differentiation-associated gene 5 (MDA5) agonist activity associated with Decoy bacteria was determined at InvivoGen (San Diego, CA) using transfected Human Embryonic Kidney (HEK293-Blue) cell line reporter gene assays. STING agonist activity was determined at InvivoGen using THP-1 human monocytic leukemia cell line reporter gene assays. Decoy10 was titrated in the range of 1x10^5^ to 5x10^8^/mL in triplicate in immune receptor and negative control assays. Cells were incubated for 16-24 hours before reporter assay. Cell line-appropriate negative control activity was subtracted from all Decoy10 results and resulting inductions greater than or equal to 2-fold were considered to represent agonist activity, as recommended by Invivogen. The Decoy10 freezing medium (vehicle) did not produce activity. The reporter gene fold-induction by the lowest saturating concentration of Decoy10 was compared to the fold-induction obtained with the lowest saturating concentration of optimal natural or synthetic positive control agonists for each receptor. InvivoGen does not test live bacteria, so comparisons to unprocessed bacteria could not be investigated at the CRO. Heat-killing of parental bacteria resulted in variable loss of TLR4 agonist activity, as assessed by the LAL assay, demonstrating that this approach was not suitable for assessment of unprocessed bacteria at InvivoGen. Therefore, the HEK293-Blue TLR4 reporter gene cell line was licensed from InvivoGen and tested at MDS using live, unprocessed parental cells and Decoy10. The results demonstrated that the PMB + GA process-mediated LPS-endotoxin activity reduction as assessed by the LAL assay was correlated with a similar reduction as determined by the TLR4 reporter gene assay (data not shown).

Induction of cytokine and chemokine secretion from human peripheral blood mononuclear cells (PBMCs) by Decoy bacteria and TLR agonists was assessed at Eurofins Panlabs (St. Charles, MO) using Luminex technology with Cytokine/Chemokine magnetic bead panels from Millipore. Frozen PBMCs from a male, Caucasian, 20 to 30-year-old, healthy volunteer were used. Assays were performed in triplicate in 96-well format with 2.5x10^5^ PBMCs in 200 µL RPMI medium, containing 2.5 mM Glutamine, 10% human serum AB (Gemini #100-318), and 1% Pen/Strep per well. Cells and compounds were incubated for 48 hours prior to analysis of supernatants. Monospecific TLR agonists were reconstituted and diluted as instructed by the manufacturer and titrated to final concentrations as follows: Poly (I:C) HMW (InvivoGen #tlrl-pic) 0.001, 1, 10, and 100 µg/mL; *E. coli* LPS (InvivoGen #tlrl-pb5lps) 10, 1x10^2^, 1x10^3^, 1x10^4^, 1x10^5^, and 1x10^6^ pg/mL; R848 (InvivoGen #tlrl-r848) 0.1, 1, 10, and 100 µg/mL; CpG oligonucleotide (ODN) 2395 (InvivoGen #tlrl-2395) 0/00005, 0.0005, 0.005, 0.05, 0.5, and 5 µM; CpG oligonucleotide (ODN) 2006 (InvivoGen #tlrl-2006) 0.005, 0.05, 0.5, and 5 µM. Levels of cytokine induction were interpolated off a standard curve using a 5-point non-linear regression analysis where the fit = (A+((B-A)/(1+(((B-E)/(E-A))*((x/C)^D))))). The interpolated data was normalized to vehicle controls or unstimulated control as appropriate. Experiments were also carried out with mouse PBMCs in mouse serum.

### 2.3 *In vivo* studies

#### 2.3.1 Formulations and dosing

Unless otherwise indicated, frozen aliquots of Decoy bacteria in freezing medium containing 12% trehalose were centrifuged at ≥3,000 x *g* for 10 minutes in a microfuge and resuspended in PBS, 2 mM MgCl2, pH 7.5 (Decoy vehicle) prior to tail vein administration of 0.1 mL to mice via a slow push. The Decoy vehicle was used as the no treatment control in some, but not all, studies and did not produce significant anti-tumor effects or toxicity. Dose-response titrations of Decoy10 or Decoy20 demonstrated that 2-3x10^8^ bacteria per dose, administered once or twice per week (two days in a row) for 2-4 weeks, generally produced optimal single agent or combination-mediated efficacy. These schedules were associated with minimal clinical signs of toxicity, generally consisting of transient 5-8% average group body weight loss relative to pre-treatment for ~2 days after dosing in the first 1-2 weeks of treatment. Less or no body weight loss was observed after treatment in subsequent weeks, likely due to the tolerance phenomenon associated with LPS (66). Frozen Decoy aliquots were used only once on the day of thawing.

Combination studies were conducted with low-dose, oral (p.o.) indomethacin (Sigma-Aldrich I7378), intraperitoneal (i.p.) anti-PD-1 antibody (BioXCell RMP1-14, BE0146), low-dose, i.p. cyclophosphamide (LDC) (Shanxi Pude Pharmaceutical Company), and/or i.p. rituximab (Roche). Indomethacin stock was prepared at 5 or 7 mg/mL in 100% ethanol and stored at -20°C. Stock was diluted 500-fold in drinking water to 10 or 14 µg/mL and the pH was adjusted, if necessary, to ≤7.2. Water bottles with indomethacin were changed daily (67). Anti-PD-1 antibody was administered i.p. at 10 mg/kg in PBS from a 2.5 mg/mL dosing solution twice per week (Q3-4 days) for two weeks. LDC was administered i.p. at 20 mg/kg in PBS from a 5.0 mg/mL dosing solution four times per week (four days in a row) for two or three weeks. Rituximab was administered i.p. at 100 µg/mouse in normal saline for injection from a 1 mg/mL dosing solution twice per week (Q3-4 days) for 3 weeks. Indomethacin, LDC, anti-PD-1, and rituximab administration was generally initiated on the day of randomization, one day before 1^st^ Decoy administration.

#### 2.3.2 Mice and tumor models

Murine syngeneic and human tumor xenograft anti-tumor studies in mice were conducted with subcutaneous (s.c.) CT26.WT murine colon carcinoma (ATCC) in ~11-week-old female BALB/c mice (Charles River Laboratories International, Inc. [CRL]) at Southern Research Institute (SRI, Birmingham, AL); with orthotopic CT26.WT-green fluorescent protein (GFP) murine colon carcinoma in 5-6-week-old female BALB/c mice (CRL) at AntiCancer, Inc. (San Diego, CA); with s.c. CT-26 murine colon carcinoma (HFK Bio-Technology Co. Ltd, Beijing, China) in 6-8-week-old female BALB/c mice; s.c. A20 murine NHL (ATCC) in 6-8-week-old female BALB/c mice (HFK Bio-Technology Co. Ltd, Beijing, China), s.c. Ramos human NHL (ATCC) in 6-8-week-old female CB17/SCID mice (Vital River Animal Technology Co., Beijing, China), metastatic Pan02 murine pancreatic carcinoma (NIH) in 6-8-week-old female C57BL/6 mice (Vital River Animal Technology Co., Beijing, China), and s.c. H22 murine hepatocellular carcinoma (China Center for Type Culture Collection) in 8-10-week-old female BALB/c mice (HFK Bio-Technology Co. Ltd, Beijing, China) at Crown Bioscience, Inc. (Beijing, China); and with s.c. H22 murine hepatocellular carcinoma in 7-week-old BALB/c female mice (Jackson Labs) at Crown Bioscience, Inc. (San Diego, CA). Mice were monitored daily for morbidity, mortality, and any clinical signs of toxicity. Individual animal body weights were generally determined daily or 5-times per week during treatment and twice per week after cessation of treatment unless otherwise indicated. Tumor growth and metastasis were monitored by palpation, bidirectional caliper measurements, GFP fluorescence imaging, and/or post-mortem examination as appropriate. Mice were humanely sacrificed based on deteriorating condition, if in distress, if moribund, with excessive s.c. tumor ulceration, ≥25% body weight loss, or tumor volume ≥2,500 or 3,000 mm^3^, depending on the model. All *in vivo* experiments were approved by and carried out under the auspices of an Institutional Animal Care and Use Committee (IACUC).

Most s.c. tumor model studies were initiated when the average tumor volume was approximately 200 mm^3^. A few studies were initiated with smaller s.c. tumors and one study was carried out with non-established tumors, as indicated. Tumor volumes (V) in s.c. studies were determined with caliper measurements (V=(LxWxW)/2). *In vivo* data was analyzed for statistical significance by two-tailed t-test, log-rank test (Kaplan-Meier survival curves) and/or non-parametric Mann-Whitney U test, using Provantis (v8, Instem Life Sciences Systems), SPSS (v18.0, Statistical Product and Services Solutions, IBM) or GraphPad Prism software. Most *in vivo* studies were followed for ≥70 to 140 days post tumor cell implant in order to determine if regressions were durable.

The CT26.WT-GFP orthotopic model at AntiCancer (San Diego) was implanted by anesthetizing the animals with a mixture of ketamine, acepromazine and xylazine. The surgical area was sterilized with iodine solution and alcohol. A vertical incision of approximately 1.5 cm was made on the lower left abdomen. The cecum was exteriorized from the abdominal cavity. The serosa on the implantation site was removed using two forceps. Two pieces of tumor fragments from an s.c., tumor were sutured on the cecum with sterile 8-0 surgical sutures (nylon). The cecum was then returned to the abdominal cavity. The incision on the abdominal wall was closed with sterile 6-0 surgical sutures in one layer. All procedures of the operation described above were performed under a 7x magnification microscope (Olympus). Mice were randomized based on animal weight and best general health (7 mice per group). Liquid clinical grade 5-fluorouracil (5-FU) was diluted with normal saline to 1 mg/mL and 10 mL/kg was administered i.p. to achieve a final dose of 10 mg/kg. Body weights were measured twice per week.

An intrasplenic, metastatic pancreatic carcinoma model was used at Crown Bioscience, Inc. (Beijing). Mice were anesthetized by intraperitoneal injection of 1% pentobarbital sodium according to body weight (10 mL/kg). After mice reached deep anesthesia, an incision was made at the site of the spleen and the spleen was exposed, then Pan02 cell suspensions from an exponential phase monolayer culture (3x10^6^ cells) in 25 µL PBS with matrigel (1:1) were inoculated into the spleen using an insulin syringe, followed by slight pressure on the spleen for 20-30 seconds. The abdominal wall was closed using a No. 6 suture and then sterilized with povidone iodine solution. The mice were kept warm until they recovered from anesthesia. Mice were assigned to groups of 7 using a randomized block design starting with homogeneous blocks based on weight. A development study was carried out to determine the optimal tumor cell inoculation number. Gemcitabine (Carbosynth China Limited, ND093431401) was administered i.p. (10 mL/kg solution in normal saline) at 50 mg/kg twice per week for 7 weeks. Body weights were measured before and at least daily for three days after Decoy20 treatment. Metastasis from the spleen to the liver and pancreas was assessed and confirmed by post-mortem necropsy.

Plasma cytokine and chemokine analysis with the s.c. syngeneic H22 HCC model was conducted at Crown Bioscience, Inc. (Beijing) with 5 mice per group having an average s.c. tumor volume at randomization of 199 mm^3^. The following day (Day 1), mice were untreated or treated with 10 µg/mL indomethacin in drinking water QDx7, 10 mg/kg anti-PD-1 antibody i.p. on Days 1 and 4, 2x10^8^ Decoy10 i.v. once on Day 2, or all possible two-way and the three-way combinations. Plasma was prepared from satellite groups of 5 mice 6 and 24 hours after study initiation (no treatment), after the first administration of each compound as single agent or in the various combinations. Whole blood (300~500 uL) was obtained by jaw vein puncture into EDTA-K2 anticoagulation tubes. Tubes were inverted several times, placed on ice, then centrifuged at 8,000 rpm for 5 min. The supernatant (~150 uL plasma) was placed into 1.5 mL epoxy resin (EP) vials and stored at -80°C. Animals were euthanized by cervical dislocation after whole blood collection. Cytokine and chemokine analysis was carried out with a Luminex 200TM instrument using a Milliplex 32-Plex MAP Mouse Cytokine/Chemokine Magnetic Bead Panel from EMD Millipore. Statistically significant induction, relative to the control group, was determined by first using a Bartlett test to check homogeneity of variance and normality, followed by a non-parametric Mann-Whitney U test. Statistically significant inductions or reductions relative to no treatment are reported. Results with 3 analytes (IL-3, IL-4, IL-13) were excluded because almost all values were below the lower limit of quantitation (LLoQ) and all differences in expression relative to no treatment were less than two-fold, reflecting differences in reported lower limit of quantitation (LLOQ) between assays.

Tumor gene expression analysis with the s.c. syngeneic H22 HCC model was conducted with 6 mice per group in the same experiment described above for plasma cytokine/chemokine analysis. Mice were untreated or treated as described above for plasma cytokine/chemokine analysis. Tumor volume was determined 3 and 7 days after initiation of dosing (Days 4 and 8). Mice were then sacrificed by cervical dislocation, tumors were isolated, cut into maximum 30 mg samples, placed into RNAlater (Fisher Scientific), stored at 4°C overnight, removed, frozen at -80°C and then shipped to WuXi AppTec (Shanghai) for nCounter PanCancer Mouse IO360 nanoString gene expression analysis (nanoString-XT-CSPS-MIO360-12). Analysis of twenty custom genes was added, including *BTK*, *Cd180*, *Ifnar2*, *Ifnb1*, *IL12a*, *IL12b*, *Jun*, *Map2k1*, *Map2k2*, *Mapk11*, *Mapk12*, *Mapk13*, *Mapk14*, *Nod1*, *Peli1*, *Tlr11*, *Tlr12*, *Tlr13*, *Tlr6*, and *Traf6* (**Supplementary Table S1**). RNA was extracted from the tissues with the RNeasy Mini Kit (Qiagen) and quantified by NanoDrop and Qubit. Hybridization was carried out with 50 ng purified RNA. Data was analyzed by nSolver 4.0 software and packages from R/Bioconductor, including quality control (QC) by Image QC, Binding Density, Positive Control Linearity, and Positive Control Limit of Detection QC. All samples passed QC analysis. Background Correlation and Linearity was determined by negative and positive spike-ins. Normalization was based on house-keeping genes by the Median of Ratio Method. Variance analysis was by Principal Component Analysis (PCA) and Hierarchical Clustering (HC) analysis. Differential Gene Expression Analysis was by Wald Test method, and p-values were corrected for multiple testing with Benjamini-Hochberg method. Functional enrichment analysis was based on KEGG and MSigDB databases using the clusterProfiler package. Gene expression analysis was also carried out by nanoString (Seatle, WA). P-values were adjusted using Benjamini and Hochberg False Discovery Rate (FDR) adjustment to avoid multiple comparison issues.

Immune cell pre-depletion mechanism of action studies were conducted at Crown Bioscience, Inc. (Beijing) with the A20 murine NHL model by treating 10 mice per group i.p. with 40 µL anti-asialo GM1 antibody (FujiFilm Wako #986-10001, NK cell depletion), 125 µg anti-mouse CD4 antibody (BioXCell Clone GK1.5 #BP0003-1, CD4 depletion) and/or 125 µg anti-mouse CD8 antibody (BioXCell Clone 2.43 #BP0061, CD8 depletion) Q5Dx9 starting 5 days prior to tumor cell inoculation. Mice were treated 4 times prior to randomization and 5 times after initiation of study agent treatment. Groups of 4 (out of 10 each) untreated or antibody-treated mice were removed at randomization, and cell suspensions were prepared from spleens and analyzed for immune cell content by fluorescence activated cell sorting (FACS) in order to determine % immune cell depletion.

#### 2.3.3 Acute toxicity models

Rabbit rectal temperature pyrogenicity studies (United States Pharmacopeia (USP<151>) were conducted with New Zealand White rabbits (4 animals per group) at Pacific BioLabs (Hercules, CA). Decoy vehicle, live ATCC 13070 bacteria or Decoy10 at various concentrations, diluted in sterile sodium chloride for injection to 10 mL at 37°C, were administered by slow i.v. injection via the marginal ear vein. Rectal temperatures were recorded 30 minutes prior to dosing, and every 30 minutes for 3 hours after dosing. The minimum concentrations of ATCC 13070 bacteria and Decoy10 bacteria required to increase rectal temperature by 1°C, relative to the vehicle control, were compared to determine if there was a difference in pyrogenicity.

Single dose, acute LD_50_/LD_100_ assessment was carried out with 6-8-week-old female BALB/c mice (CRL) at Molecular Diagnostic Services (San Diego). Single doses of unprocessed, live bacteria and Decoy10 were administered i.v. via the tail vein at doses ranging from 1x10^8^ to 3x10^10^ (3 mice per group).

## 3 Results

### 3.1 Production, characterization and *in vitro* immune activation by Decoy bacteria

Coley’s Toxin’s was a mixture of heat-killed, pathogenic, Gram-positive (*Streptococcus pyogenes*) and Gram-negative (*Serratia marcescens*) bacteria. The Gram-positive component was found to contribute minimally to anti-tumor efficacy and there is no reason to believe that pathogenicity was required for anti-tumor efficacy, so single laboratory strains of non-pathogenic, Gram-negative *E. coli* were used for the current approach (17).

LPS-endotoxin activity was reduced by treating live bacteria with PMB under conditions to prevent lysis. This Gram-negative antibiotic lyses bacteria by binding tightly and rather specifically to LPS in the outer membrane, sterically interfering with replicative elongation and inducing permeabilization, leading to cell death (68). A side-effect, unrelated to the antibiotic activity, is potent neutralization of LPS-endotoxin activity, as measured by the Limulus amebocyte lysate (LAL) assay (69, 70). Studies were conducted to determine the optimal bacteria concentration, PMB concentration, buffer conditions and incubation time to obtain at least 90% reduction in LAL activity, without cell lysis, which required incubation to be conducted at reduced temperature (~4°C) in the presence of excess divalent cations (MgCl_2_). Unbound PMB was removed by centrifugation-mediated washing.

The PMB-treated bacteria were killed and stabilized by incubation with glutaraldehyde (GA), followed by removal of unreacted glutaraldehyde by centrifugation-mediated washing. GA has been used to prepare vaccines and is an FDA-accepted excipient (71). A reduction of LPS-endotoxin activity by at least 90% was predicted to reduce i.v. toxicity and this was confirmed by assessment of pyrogenicity in rabbits, and by acute (single dose) lethality in mice with a Decoy10 batch exhibiting 92% reduction in LPS-endotoxin activity. The standard pyrogenicity threshold for i.v.-administered live, parental bacteria in rabbits was 3x10^4^, but was 30-fold higher (9x10^5^) for Decoy10 (**Table 1**). The acute i.v. LD_100_ dose for live, parental bacteria in mice was 1x10^10^, but was 3x10^10^ for Decoy10 (the doses tested produced either 100% survival or 100% lethality).

**Table 1:** Effects of PMB + GA treatment on bacterial viability, LPS-endotoxin activity and pyrogenicity.

Product	Viability	LPS-Endotoxin Activity EU/10^6^ Bacteria (Ave ± SEM)	Pyrogenicity Threshold (Rabbit Assay)^1^
Untreated ATCC Bacteria^2^	56-100%	96.1 ± 17.4	3x10^4^ Bacteria
Decoy10^2^	0%	3.7 ± 0.7 (Ave 96.1% Reduction)	9x10^5^ Decoy10 (97% reduction)
Untreated CGSC Bacteria^3^	55-100%	95.9 ± 14.4	
Decoy20^3^	0%	3.1 ± 1.0 (Ave 96.7% Reduction)	

The bacteria were grown, treated to produce Decoy10, and the assays were carried out as described under Materials and Methods.

^1^Carried out with one batch of ATCC bacteria/Decoy10.

^2^Data for 7 independent batches of ATCC bacteria/Decoy10.

^3^Data for 4 independent batches of CGSC bacteria/Decoy20.

**Figure 1:**
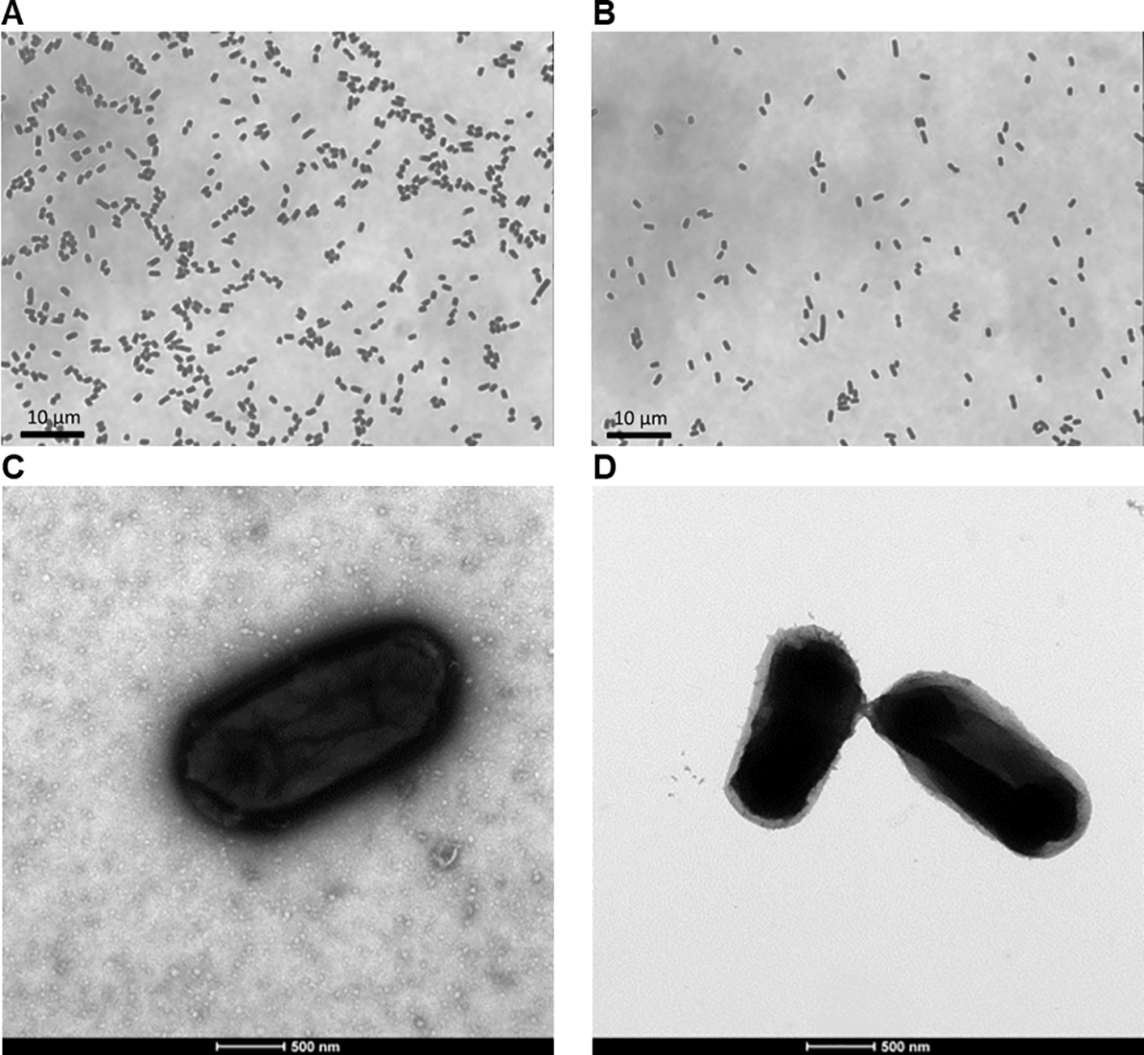
Light and electron microscope images of untreated ATCC 13070 bacteria and Decoy10. The bacteria were grown, treated to produce Decoy10, and processing were carried out as described under Materials and Methods. Grey-scale light microscope images of heat-fixed and crystal violet-stained, untreated ATCC 13070 bacteria **(A)** and Decoy10 **(B)** at ~1,000 magnification. Transmission electron microscope images of 2% uranyl acetate negatively-stained, untreated ATCC 13070 bacteria **(C)** and Decoy10 **(D)** at 23,000 magnification.

Two different diaminopimelic acid (DAP)-dependent strains of non-pathogenic *E. coli* were processed to produce Decoy10 and Decoy20. Light and electron microscope (EM) photographs of stained, untreated ATCC 13070 bacteria and Decoy10 (**Figure 1**) demonstrated that Decoy10 are intact and relatively monodisperse. The EM images suggest that the Decoy manufacturing process may strip the polysaccharide capsule from the cell surface (72). In addition to killing, stabilization of the bacteria by GA via cell surface protein crosslinking is predicted to enhance initial stability after i.v. administration, facilitating uptake, activation of and clearance by immune cells. Stabilization was confirmed by demonstrating that Decoy10 is highly resistant to sonication-mediated disruption, relative to untreated cells (**Supplementary Figure S1**). Decoy10 was also resistant to mechanical (glass bead)-mediated disruption (data not shown).

Gram-negative bacteria have been reported to contain TLR1,2,3,4,5,6,7,8,9 as well as NOD and STING agonists (73, 74). Due to the overlapping nature of the TLR, NOD and STING signal transduction pathways in immune cells, individual immune receptor agonist activity was assessed with Decoy10 using the InvivoGen reporter gene assay panel. Individual TLRs or NOD are transfected into a human embryonic kidney (HEK) cell line, or the THP-1 monocytic leukemia cell line for STING agonist analysis, in conjunction with an NF-κB (TLR, NOD) or interferon regulatory factor (IRF) (STING) reporter gene readout. Putative agonists are incubated at multiple concentrations with the cells for 16-24 hours, and activity is compared to an optimal purified or synthetic positive control agonist. The kidney cells are not immune cells and may not process intact bacterial cells in the same way as immune cells. In addition, the assay does not detect TLR signaling via the IRF pathway. Due to these limitations and the use of a single time-point, these assays should be considered qualitative read-outs of whether an agonist is present in the test agent.

Decoy10 exhibited agonist activity (>2-fold induction) for TLR2 (including TLR1/2 and TLR2/6), TLR3, TLR4, TLR5, TLR8, TLR9, NOD2 and STING, with maximum average activity ranging from 14% to 98% of the maximum purified/synthetic positive control activity. TLR3 and TLR5 activity consistently saturated at ≤20% of their respective positive controls (**Table 2**). TLR7, NOD1, MDA-5, RIG-I, Mincle, Dectin-1a, and Dectin-1b agonist activity was assessed once with Decoy10 and was not detected (<2-fold induction). Decoy10 was also assessed once with several of the equivalent mouse immune receptor reporter gene assays and a similar pattern of agonist activity was observed, although with lower levels of induction (data not shown).

**Table 2:** Reporter gene assay immune receptor agonist activity associated with Decoy10.

Human Immune Receptor Cell Line	Decoy10-Induced Percent of Saturating Positive Control Activity	Positive Control
TLR2/1	104/97	Pam3CSK4
TLR2/6	37/40	FSL-1
TLR2 (2/1 + 2/6)	107/96	HKLM
TLR3	16/20	Poly(I:C) HMW
TLR4	94/86	*E. coli* K-12 LPS
TLR5	12/15	*S. typhimurium* flagellin
TLR7	0/0	CL307
TLR8	41/60	TL8-506
TLR9	65/49	CpG ODN 2006
NOD1	0	C12-iE-DAP
NOD2	16/38	L18-MDP
STING	7/16/41	2’3’-cGAMP
Dectin-1a	0	*S. cerevisiae* β-glucan
Dectin-1b	0	Zymosan Depleted
Mincle	0	Trehalose-6, 6-dibehenate
RIG-I	0	Poly(I:C) HMW/LyoVec
MDA-5	0	5’ppp-dsRNA/LyoVec

Assays were carried out as described under Materials and Methods. Results for 1-3 independent assays for each receptor are listed. Decoy10-mediated inductions were 3.0 to 3.6-fold for TLR3 and TLR5, 3.1 to 8.2-fold for NOD2, and ranged from 7.4 to 36.9-fold for all other assays. Variability in the STING results was partly due to variability in saturating positive control activity between experiments.

Decoy10 dose response experiments revealed that TLR2 and TLR4 agonist activity saturated at <5x10^6^ Decoy10/mL. STING agonist activity saturated at <10^7^ Decoy10/mL, NOD2 agonist activity saturated at ~10^7^ Decoy10/mL, and TLR3,5,8, and 9 agonist activity saturated at >10^8^ Decoy10/mL, suggesting that these agonists are present at significantly lower concentration in Decoy10 than TLR2,4, STING, and NOD2 agonists.

TLR agonists induce immune responses, including both positive (anti-tumor and anti-pathogen) and negative (toxic) responses, via both direct intracellular signaling and induction of secretion of cytokines and chemokines. One possibility was that the reduced i.v. pyrogenicity and toxicity exhibited by Decoy10 relative to unprocessed parental cells is due to a reduction in immune cell secretion of cytokines and chemokines, due to the significantly reduced LPS-endotoxin TLR4 agonist activity. Surprisingly, despite the reductions in LPS-endotoxin activity, acute toxicity and pyrogenicity, Decoy10 and Decoy20 were found to induce secretion of similar or higher levels of all but one tested cytokine/chemokine from human PBMCs, relative to the same dose of unprocessed parental bacterial cells (**Table 3**). The only exception was IFN-γ, where untreated bacteria induced about 2-fold higher peak concentrations than Decoy bacteria, although the IFN-γ levels induced by Decoy bacteria are still extremely high. Decoy10 and Decoy20 also induced higher levels of secreted cytokines and chemokines than commercially available mono-specific TLR agonists (**Table 4**). This is not surprising, due to the presence of multiple immune receptor agonists in the Decoy bacteria.

**Table 3:** Cytokine and chemokine secretion by human PBMCs *in vitro* induced by untreated or Decoy bacteria.

Cytokine/Chemokine Secretion by Human PBMCs *In Vitro*					
Cytokine or Chemokine	Bacteria or Decoy Dose/mL	Untreated ATCC Bacteria	Decoy10	Untreated CGSC Bacteria	Decoy20
		4,470 EU/10^8^ Cells	221 EU/10^8^ Cells	12,408 EU/10^8^ Cells	118 EU/10^8^ Cells
		Cytokine or Chemokine Peak (pg/mL/mean of triplicates + (%CV))			
GM-CSF	1x10^8^	1,094 (22)	1,197 (2)	1,493 (34)	1,695 (23)
IFN-α_2_	1x10^8^	16 (61)	6 (62)	20 (33)	0
IFN-γ	1x10^8^	107,866 (20)/(175,866 (7)	91,475 (12)	166,795 (12)	75,530 (14)
IL-1β	1x10^7^	11,976 (9)	17,651 (10)	10,571 (4)	19,232 (2)
IL-2	1x10^8^	3 (13)	8 (55)	1 (43)	4
IL-6	1x10^6^	78,422 (1)	98,534 (9)	58,656 (15)	89,332 (42)
IL-8	1x10^5^	126,942 (20)	166,769 (16)	127,461 (5)	145,921 (2)
IL-10	1x10^7^	6,970 (3)	7,620 (3)	5,223 (11)	5,882 (3)
IL-12p70	1x10^7^	176 (14)	528 (7)	125	428 (37)
IL-17A	1x10^8^	8 (34)	13 (40)	5	7 (4)
IL-23	1x10^6^	<8	119 (11)	<8	176 (24)
TNF-α	1x10^7^	49,782 (11)	77,919 (13)	41,035 (5)/50,992 (12)	99,247 (16)

Assays were carried out as described under Materials and Methods (EU = Endotoxin Units). Bacteria samples were titrated in 10-fold increments from 10 to 1x10^8^ per mL and incubated with PBMCs for 48 hours before Luminex analysis of supernatants. Peak average triplicate cytokine/chemokine secretion values (reported with % Coefficient of Variation) occurred at the same untreated bacteria and Decoy dose for all except two analytes, where the peak with untreated bacteria was observed at a higher concentration (peak value indicated as second number).

**Table 4:** Cytokine and chemokine secretion by human PBMCs *in vitro* induced by untreated bacteria, Decoy bacteria or monospecific TLR agonists.

Cytokine/Chemokine Secretion by Human PBMCs *In Vitro*						
	TLR3 Agonist	TLR4 Agonist	TLR7/8 Agonist	TLR9 Agonist CpG2395/CpG2006	Multiple TLR, NOD2, STING Agonist	Multiple TLR, NOD2, STING Agonist
Cytokine or Chemokine	Poly(I:C)	*E. Coli* LPS	R848		Decoy10	Decoy20 (1 Exp)
	Cytokine or Chemokine Peak (pg/mL Two Experiment Ave ± SD or 1 Exp + (%CV))					
GM-CSF	3 ± 1	326 ± 70	161 ± 35	0/0	1,271 ± 35	1,695 (23)
IFN-α_2_	139 ± 30	4 ± 1	5 ± 3	4/2	9 ± 4	0
IFN-γ	272 ± 34	35,231 ± 2,741	77,209 ± 21,630	8/7	82,898 ± 12,130	75,530 (14)
IL-1β	113 ± 11	10,283 ± 2,664	12,936 ± 982	12/17	22,010 ± 2,054	19,232 (2)
IL-2	23 ± 9	3 ± 1	1 ± 1	0/1	8 ± 0	4 (89)
IL-6	817 ± 110	81,891 ± 13,065	36,640 ± 1,846	375/241	46,964 ± 24,310	89,332 (42)
IL-8	1,288 ± 233	107,276 ± 51,170	165,292 ± 69,518	0/2,435	111,028 ± 78,830	145,921 (2)
IL-10	21 ± 6	3,864 ± 151	991 ± 24	465/374	7,943 ± 129	5,882 (3)
IL-12p70	14 ± 2	71 ± 18	181 ± 34	3/4	299 ± 108	428 (37)
TNF-α	302 ± 46	25,181 ± 335	38,298 ± 2,312	0/65	70,644 ± 3,430	99,247 (16)

Assays were carried out as described under Materials and Methods. Monospecific TLR agonists were formulated and titrated as recommended by manufacturer (doses in Materials and Methods). Peak inductions (pg/mL) are reported from the average of two independent triplicate experiments (± SD), except for Decoy20, which is reported from one triplicate experiment with % Coefficient of Variation.

Human PBMCs in human serum are one to four orders of magnitude more sensitive than mouse PBMCs in mouse serum to induction of cytokine secretion by both LPS and heat-killed *E. coli* (75). Similar results were obtained with Decoy10 and Decoy20, with little or no IFN-γ or TNF-α secretion observed with mouse PBMCs (Newman M.J., unpublished results), suggesting that much lower doses of Decoy bacteria may be required for immune activation in humans, relative to mice.

### 3.2 Single agent *in vivo* anti-tumor activity of Decoy bacteria (colorectal and pancreatic carcinoma models)

**Figure 2:**
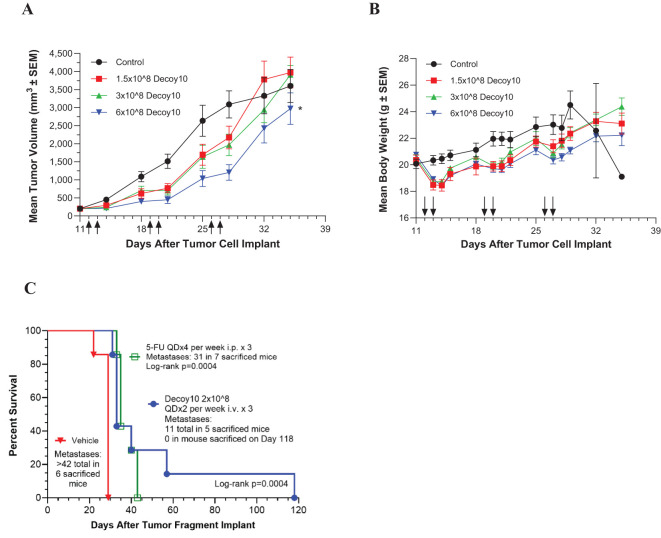
Decoy10 inhibits the growth of s.c. CT-26 murine colorectal carcinoma, and extends survival and inhibits metastasis of an orthotopic CT-26 model. The experiments were carried out as described under Materials and Methods. **(A)** Tumor cells (2x10^5^ in PBS) were implanted s.c. on Day 0 and i.v. treatment with Decoy10 (QDx2 per week for 3 weeks)) with 8 mice per group was initiated one day after randomization on Day 11 when the average tumor volume was 202 mm^3^. Arrows denote dosing days. Body weight was measured 4 times per week. The highest Decoy10 dose group produced statistically significant tumor growth inhibition relative to the control (untreated) group (*Log-rank p=0.023). **(B)** Mean body weights recorded during the study are presented. There were no treatment-related deaths or requirements for dosing holidays. **(C)** CT-26-GFP tumor fragments were surgically implanted on the cecum. Randomization and treatments were initiated 5 days after tumor fragment implant (7 mice per group), with 5-FU starting the same day and Decoy10 starting one day after randomization. Body weights were measured twice per week. Average group body weight loss with Decoy10 treatment relative to randomization was only observed once on Day 15 (4.0%).

**Figure 3:**
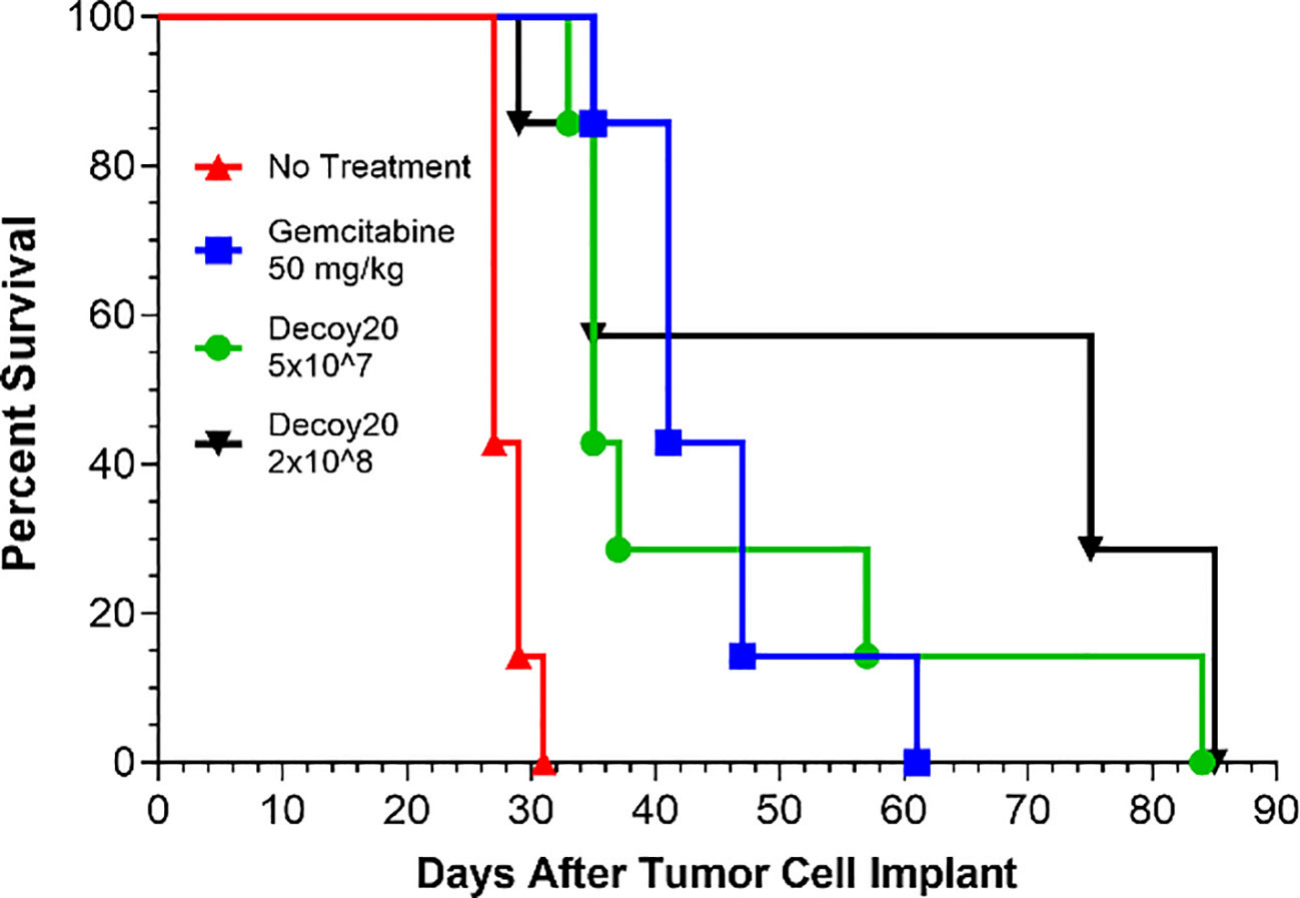
Decoy20 extends survival in a metastatic, murine pancreatic carcinoma model. The experiment was carried out as described under Materials and Methods. Randomization and treatments were initiated 5 days after tumor cell implant (7 mice per group), with i.p. Gemcitabine at 50 mg/kg QDx4 per week for 3 weeks starting the same day and i.v. Decoy20 at 5x10^7^ or 2x10^8^ per mouse QDx2 per week starting one day after randomization. Median survival was 27 Days (No Treatment), 41 Days (Gemcitabine), 35 Days (5x10^7^ Decoy20), and 75 Days (2x10^8^ Decoy20). All median survival increases were statistically significant relative to No Treatment by Log-rank analysis (p<0.001). Body weights were measured before and at least daily for three days after Decoy20 treatment. Maximum transient average group weight loss was 8.2% and 9.8% for the low and high Decoy20 doses, respectively, during the first week of treatment, and 2.1% and 2.6% in the second week of treatment, with no weight loss in the third week of treatment, demonstrating toxicity tolerance with repeat dosing.

LPS-endotoxin-attenuated and killed Decoy10 and Decoy20 bacteria were assessed for *in vivo* anti-tumor activity at multiple CROs and in multiple models. Decoy bacteria were administered i.v. (via the tail vein) once or twice per week (mostly QDx2) for 2 to 6 weeks in most experiments. **Figure 2A** demonstrates inhibition of established, sub-cutaneous (s.c.) CT-26 colorectal carcinoma growth by Decoy10. The highest dose of Decoy10 produce a statistically significant inhibition of tumor growth relative to control (no treatment). Maximum transient 2-3 day average group body weight loss relative to the day before initiation of treatment (all treated groups) was 9-11% observed in the first week of treatment, 2-5% in the second week of treatment, and 0-2% in the third week of treatment, with an indication of tolerance, particularly at the lowest and middle doses (**Figure 2B**). The highest dose also produced transient observations of ruffled fur, but no treatment related deaths or required dose holidays. Single agent anti-tumor activity and inhibition of metastasis was observed with Decoy10 at a second CRO using an orthotopic CT-26-GFP colorectal carcinoma model (**Figure 2C**). Single agent Decoy20 activity was also observed with a metastatic pancreatic carcinoma model (Pan02) (**Figure 3**). Statistically significant inhibition by Decoy10 or Decoy20 with this metastatic pancreatic carcinoma model, via Log-rank analysis, was observed in four independent experiments.

### 3.3 Combination therapy-mediated durable tumor regression, induction of immunological memory, and mechanism of action of Decoy bacteria (HCC and NHL models)

**Figure 4:**
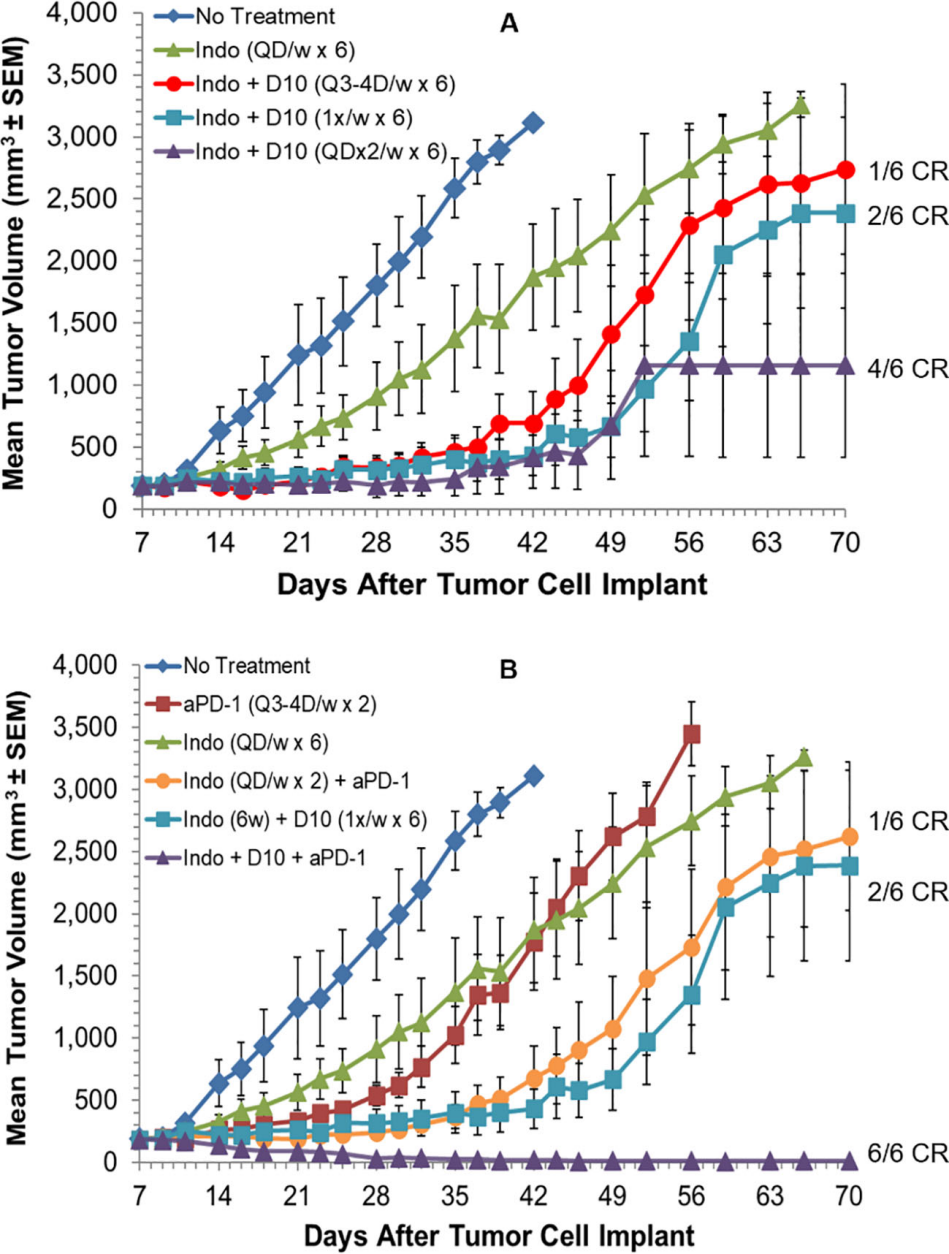
Decoy10 synergizes with indomethacin and anti-PD-1 to regress established tumors in the s.c. murine H22 hepatocellular carcinoma model. The experiment was carried out as described under Materials and Methods. Tumor cell implantation was carried out s.c. with 2x10^6^ H22 cells in PBS. Mice were randomized and indomethacin (Indo) treatments were initiated on Day 7 when the average tumor volume was 187 mm3. **(A)** Indo was administered QD for 6 weeks at 10 µg/mL in the drinking water (no regressions, Log-rank p=0.007 vs no treatment). Decoy10 (D10) was not tested alone in this experiment but produced slight statistically significant tumor growth delay without producing any regressions at QDx2 per week (**Figure 5**). D10 was administered starting one day after Indo. Indo + QDx2 D10 per week produced optimal combination synergy (4/6 CR, Log-rank p=0.018 vs indomethacin). All regressions in this part of the study were durable until termination on Day 91. Transient, average group body weight loss during each week of treatment, relative to randomization day, for the Indo + QDx2 per week D10 group was 9.5%, 7.7%, 4.1%, 1.2%, 0.9%, and 0, respectively. **(B)** Anti-PD-1 was tested alone (Q3-4 days per week for 2 weeks) (Log-rank p=0.002 vs no treatment), with Indo administered QD for 2 weeks, and with Indo (6 weeks) + the three different D10 schedules in part A (only the optimal triple combination is shown). Indo + anti-PD-1 produced 1 durable CR, Indo + once per week D10 produced 2 durable CRs. With the triple combination, once per week D10 produced the best result (6/6 CRs, with 5/6 durable to study termination on day 91) (Log-rank p=0.018 vs Indo + D10, and p=0.004 vs Indo + Anti-PD-1. Transient, average weekly group weight loss with this triple combination (8.8%, 6.1%, 3.7%, 2.2%, 5.2%, and 0.8%) was similar to the weight loss observed with the optimal double combination of Indo + Decoy10 in part **(A)** The QDx2 per week D10 triple combination schedule produced 3/6 CRs (1 durable) and the Q3-4 day per week D10 triple combination schedule produced 4/6 CRs (3 durable) (not shown).

Combination and mechanism of action studies were conducted *in vivo* with two additional syngeneic models and one human tumor xenograft model. Initial studies were done with the established, s.c., murine H22 hepatocellular carcinoma model. The first potential synergy partner tested was an oral, low-dose, non-steroidal anti-inflammatory drug, indomethacin. Indomethacin has previously been reported to synergize with anti-tumor immunotherapies, including anti-PD-1, to regress tumors. This may be mediated, in part, by inhibition of myeloid-derived suppressor cell (MDSC) and other immunosuppressive activities, via reduction of prostaglandin biosynthesis (67, 76–78). Indomethacin was administered daily at 10 µg/mL in drinking water (changed daily). Mice have been reported to drink an average of ~5.0 mL water per day, which would translate to an indomethacin dose of 2.5 mg/kg/day for a 20 g mouse, or 14 mg per day for a 70 kg human, based on allometric scaling. This dose did not produce any weight loss or other clinical signs of toxicity when administered QD to mice for up to 7 weeks. Indomethacin treatment for 6 weeks produced a statistically significant delay of tumor growth relative to no treatment (Log-rank p=0.007) but resulted in no tumor regressions (**Figure 4A**).

Single agent Decoy10 occasionally produced slight tumor growth delay in this model when administered QDx2 per week, but no regressions (not tested in this experiment). Indomethacin was tested in combination with three different schedules of i.v. Decoy10. The optimal Decoy10 schedule was QDx2 (two days in a row) per week, starting one day after initiation of indomethacin treatment. This combination produced 4/6 durable, complete regressions or responses (CR), and was statistically significant relative to single agent indomethacin (Log-rank p=0.018), suggesting a synergistic interaction. Similar results were obtained with Decoy20 (3/6 durable regressions) (data not shown). Twice per week Decoy10 (Q3-4 days) produced 1/6 durable regressions and once per week Decoy10 produced 2/6 durable regressions in combination with indomethacin. All regressions were durable through experiment termination on Day 91. Transient average group body weight loss of 9.5% relative to randomization was observed with the optimal combination after treatment in the first week, and decreased with each successive week of treatment, demonstrating tolerance. Four weeks of Decoy and indomethacin treatment were generally sufficient to produce maximum durable regressions. Indomethacin occasionally produced one single agent regression (out of six mice) in the H22 model at the CRO used for the experiment in **Figure 4A**, and in 2/6 mice at a second CRO. This was usually associated with a tumor volume of ≤150 mm^3^ at initiation of treatment.

Anti-PD-1 was tested in the H22 model as a single agent, in combination with indomethacin, and in combination with indomethacin + Decoy10, using the three Decoy10 schedules reported above (**Figure 4B**). Anti-PD-1 was tested using a standard dose and schedule of Q3-4 days twice per week for two weeks, producing tumor growth delay without regressions. Combination of anti-PD-1 with daily indomethacin for 2 weeks produced one durable regression. When the 6-week p.o. indomethacin + once per week i.v. Decoy10 regimen, that produced 2/6 regressions in **Figure 4A**, was combined with anti-PD-1 treatment, the triple combination produced 6/6 regressions, 5 of which were durable through Day 91. The triple combination result was statistically significant compared to either the indomethacin + Decoy10 or indomethacin + anti-PD-1 combination (Log-rank p values of 0.018 and 0.004, respectively). Weight loss induced by the optimal triple combination schedule was similar to single agent Decoy10 and the double combination with indomethacin. There were no other signs of toxicity.

**Figure 5:**
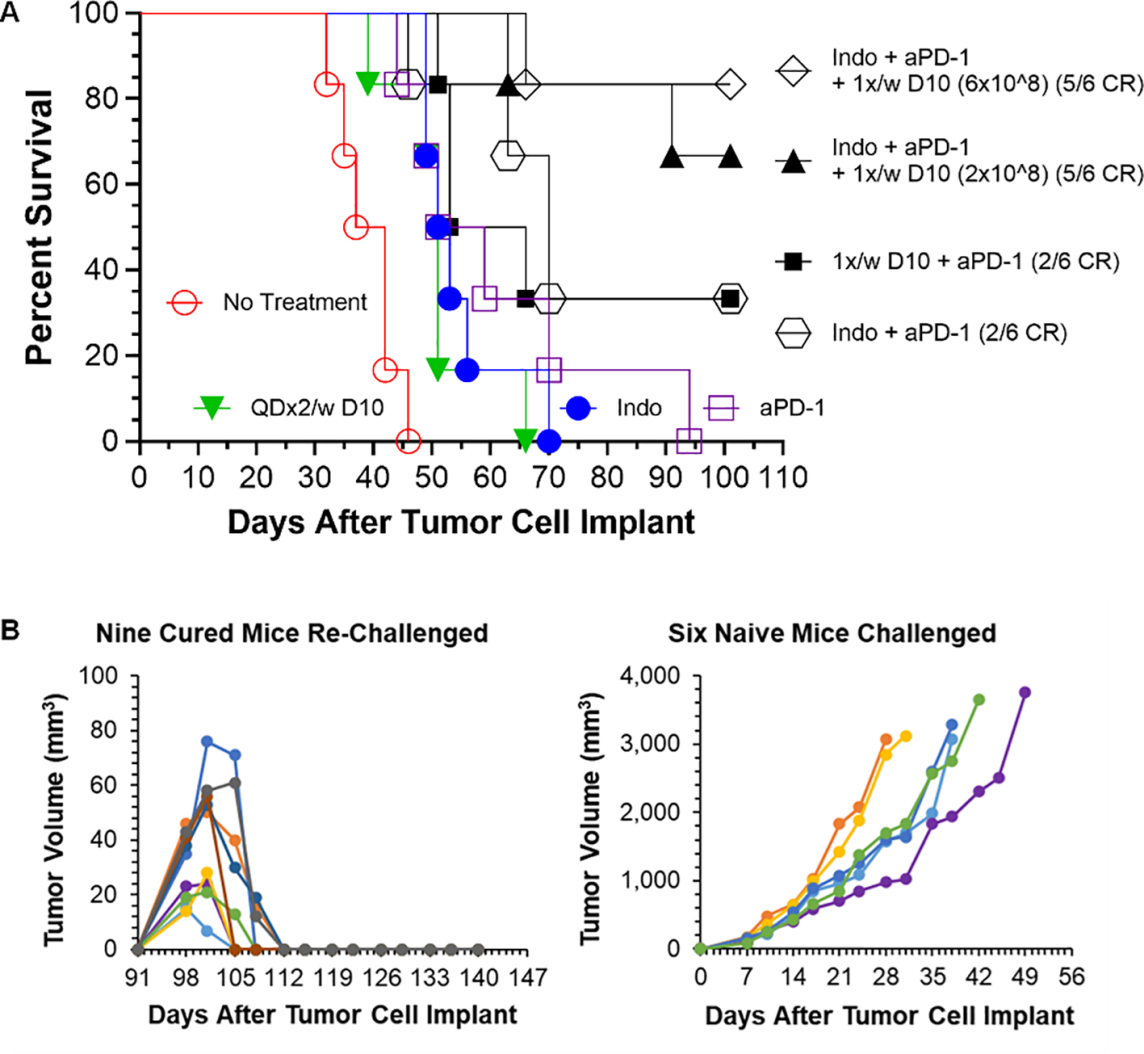
Decoy10 synergizes with anti-PD1 to regress established tumors in the s.c. H22 hepatocellular carcinoma model and the triple combination induces immunological memory. The experiment was carried out as described under Materials and Methods. Implantation was carried out with 2x10^6^ H22 cells in PBS. Treatments were initiated on Day 7 when tumors averaged 194 mm^3^ with 6 mice per group. Indomethacin (Indo) was administered p.o. at 10 µg/mL in drinking water QD for 6 weeks starting on Day 7. Decoy10 was administered i.v. at 2x10^8^ QDx2 per week as single agent or once per week in combinations, both for 6 weeks starting on Day 8. Anti-PD-1 (aPD-1) was administered i.p. at 10 mg/kg Q3-4 days per week for 2 weeks starting on Day 7. **(A)** All treatments (single and combination) produced a statistically significant enhancement of survival relative to no treatment (Log-rank p ≤ 0.006). There were no regressions with single agent treatments. The 2-way combinations each produced 2/6 full, durable regressions (to termination at Day 101) and the 3-way combinations (two different Decoy10 doses) produced 10/12 full regressions with 9 durable to termination at Day 140. The maximum transient, weekly average group body weight loss was 6.9%, 5.3%, 5.5%, 3.3%, 1%, and 0 respectively after each of the six QDx2 weekly doses of single agent Decoy10, and was 8.30%, 5.0%, 5.4%, 2.9%, 3.7%, and 0 for the triple combination with the higher dose of once per week Decoy10. Decoy10 + indomethacin was not tested in this experiment. **(B)** The nine triple combination mice with durable regressions were re-challenged with H22 cells on the opposite flank from the first tumor challenge on Day 91 (no further treatment). All rechallenge tumors started to grow and then were fully rejected demonstrating 100% immunological memory. Full tumor take was recorded in naïve mice that received the same tumor cells on the same day as the re-challenge.

The experiment in **Figure 4** was repeated in order to test combination of Decoy10 with anti-PD-1 in the absence of indomethacin, and to test if the durable regressions produced with the triple combination are associated with immunological memory (**Figures 5A, B**). Decoy10 was tested in its optimal single agent schedule (QDx2 per week), producing a slight, but statistically significant, increase in lifespan (Log-rank p=0.006) with no regressions. Indomethacin reproducibly produced a slight increase in lifespan (Log-rank p=0.001) with no regressions. Single agent anti-PD-1 also produced a slight, but statistically significant, increase in lifespan (Log-rank p=0.002) with no regressions. Combination of indomethacin for 6 weeks with anti-PD-1 for 2 weeks produced 2/6 durable regressions, and combination of anti-PD-1 with once per week Decoy10 also produced 2/6 durable regressions. QDx2 Decoy10 + anti-PD-1 produced 1/6 durable regression (not shown). The triple combination with once per week 2x10^8^ Decoy10 produced 5/6 regressions (4 durable), and the 6x10^8^ dose of Decoy10 produced 5/6 durable regressions, similar to the results seen in the experiment reported in **Figure 4B**. The transient, weekly weight loss patterns for Decoy10 were similar in the single, double, and triple combination settings in all experiments with this model. Maximum percent regressions with the triple combination setting were found to require ~4 weeks of once per week Decoy treatment in repeat experiments.

The nine triple combination-treated mice with durable regressions in **Figure 5A** (two different Decoy10 doses) were rechallenged on the opposite flank from the first tumor challenge with fresh H22 HCC cells on Day 91 (no additional study agent therapy). All of the tumors started to grow and were then fully rejected, demonstrating immunological memory in 100% of the previously treated mice. None of the regressed first tumor challenge sites or tumor re-challenge sites produced tumor regrowth up to termination on Day 141, while naïve mice challenged on Day 91 with fresh tumor cells exhibited full tumor growth (**Figure 5B**).

**Figure 6:**
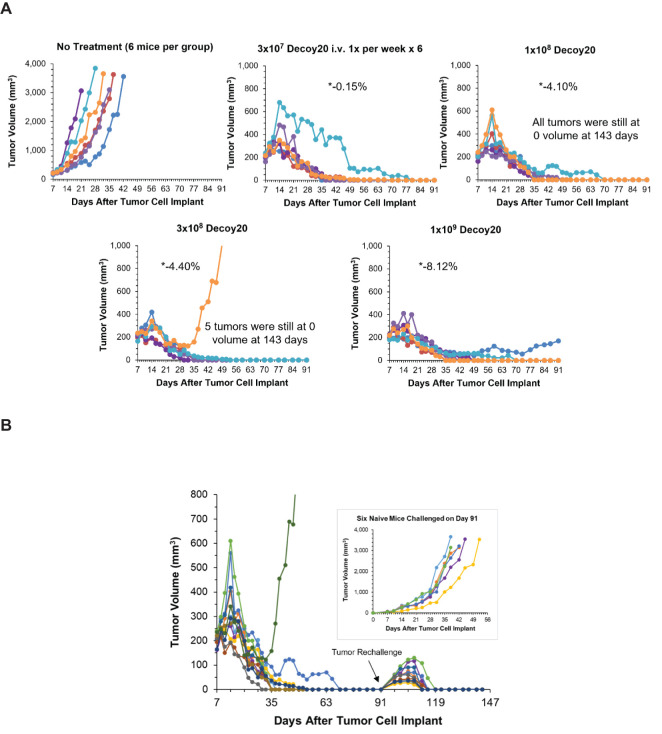
Decoy20, anti-PD-1 and indomethacin induce durable regression of established H22 HCC tumors with a Decoy20 therapeutic index of >33 and induction of immunological memory. The experiment was carried out as described under Materials and Methods. Implantation was carried out with 2x10^6^ H22 cells in PBS. Treatments were initiated on Day 7 when tumors averaged 205 mm^3^ in volume with 6 mice per group. **(A)** Indomethacin (6 weeks) and anti-PD-1 (two weeks) were administered starting on Day 7 as described in **Figures 4** and **5**. Decoy20 was administered i.v. once per week for 6 weeks starting on Day 8 at 3x10^7^, 1x10^8^, 3x10^8^, or 1x10^9^ per mouse. All doses produced 5/6 or 6/6 durable regressions (*maximum average group weight loss). **(B)** The 11 tumor-regressed mice from the two middle dose groups in part A were rechallenged on Day 91 with fresh HCC tumor cells on the opposite flank relative to the first tumor challenge. Naïve mice were challenged with the same cells on the same day. There was no further treatment.

The triple combination experiment was repeated in order to extend the results to Decoy20 and determine the Decoy therapeutic index. The study included a no treatment arm, plus a 4-dose, 33-fold titration of once per week Decoy20 in the presence of fixed concentrations of indomethacin and anti-PD-1. **Figure 6A** demonstrates that, starting with 205 mm^3^ tumors, all 4 concentrations of Decoy20 produced 5/6 or 6/6 full tumor regressions, with no transient weight loss at the lowest dose, mild, transient, maximum ~4% weight loss in the first two weeks of treatment at the two middle doses and acceptable, maximum transient 7-8% weight loss in the first two weeks of treatment at a dose of 1x10^9^ Decoy20. Tolerance with repeat dosing was observed with all doses, although to a lower extent with the highest dose. There were no treatment holidays required, no mortalities, and no clinical signs of toxicity, other than transient weight loss, demonstrating similar results with two different Decoy strains and at least a 33-fold therapeutic index. High percentage regression of established HCC tumors was observed with the triple combination of Decoy10 or Decoy20 + indomethacin + anti-PD-1 in 3 independent experiments.

The 11 tumor-regressed mice from the two middle dose groups in **Figure 6A** were rechallenged on Day 91 with fresh HCC tumor cells on the opposite flank relative to the first tumor challenge. Naïve mice were challenged with the same cells on the same day. There was no further treatment. **Figure 6B** demonstrates that tumors started to grow at the rechallenge sites in all nine rechallenged mice, but were then completely rejected, demonstrating reproducible, 100% adaptive immunological memory. The original tumor challenge sites and rechallenge sites remained tumor-free until study termination on Day 143. Challenge of naïve mice with the same tumor cells on Day 91 produced progressively growing tumors, ultimately requiring humane sacrifice. High percentage immunological memory after tumor rechallenge was observed with mice cured by the triple combination of Decoy10 or Decoy20 + indomethacin + anti-PD-1 in 2 independent experiments.

The s.c. H22 HCC model was used to evaluate *in vivo* plasma cytokine and chemokine induction by the individual therapies, p.o. indomethacin (10 µg/mL in drinking water QD), i.p. anti-PD-1 (10 mg/kg Q3-4 days per week) or i.v. Decoy10 (2x10^8^ bacteria/animal once per week), and the various combinations found to induce tumor regression. Mice were randomized into 8 groups, with an average tumor volume of 199 mm^3^, each containing 3 sub-groups. All of the possible treatment approaches or combinations, including no treatment, were carried out. Mice from each of two sub-groups from each main group (5 mice per sub-group) were sacrificed 6 and 24 hours after initiation of treatment (including no treatment), after the first single treatments, after the second compound in the first 2-way combination treatment, or after the third compound in the first 3-way combination treatment. Plasma was prepared from each mouse and a 32-plex ELISA-based cytokine/chemokine analysis was carried out. A third sub-group from each main group (6 mice each) was treated for one week (daily indomethacin, two doses of anti-PD-1 and one dose of Decoy10), with tumor volumes measured at randomization, once during the week and at the end of one week of treatment. These mice were sacrificed, then tumors were harvested, and RNA was isolated for nanoString analysis.

**Table 5** demonstrates that indomethacin produced statistically significant induction of only 2/32 cytokines/chemokines relative to no treatment, and only at 6 hours after initiation of treatment. Decoy10 produced statistically significant induction of 10/32 cytokines/chemokines and, as with indomethacin, levels were only significant compared to no treatment at 6 hours. No cytokine/chemokine induction was observed 6 or 24 hours after initiation of anti-PD-1 treatment. In the combination settings, under conditions where reproducible tumor growth inhibition and regression would ultimately be expected if treatment were continued, statistically significant induction of cytokine/chemokine expression was observed with 15 to 23 out of 32 cytokines and chemokines. Most inductions, including all with single agent Decoy10, and with the exception of the Decoy10 + anti-PD-1 combination, were only seen at 6 hours, demonstrating the transient nature of the systemic cytokine and chemokine expression. The expression of one chemokine, LIX, was reduced by treatment with Decoy10 + anti-PD-1.

**Table 5:** Tumor-eradicating combination therapy induces plasma cytokine and chemokine expression in tumor-bearing mice.

	NSAID	Decoy10	Anti-PD-1	Decoy + Anti-PD-1	NSAID + Decoy	NSAID + Anti-PD-1	NSAID + Decoy + Anti-PD-1
	Plasma prepared from mice treated as above 6 and 24 hours after single or after second/third agent in combo						
Cytokine/Chemokine	Statistically Significant Cytokine/Chemokine Induction Relative to No Treatment (at 6 and 24 hours)*						
Eotaxin	**			6			6
G-CSF				6/24	6	6	6
GM-CSF				6/24	6	6	6
IFN-gamma				6			
IL-1alpha		6		6/24	6	6	6
IL-1beta				6	6	6	6
IL-2				6/24	24	24	24
IL-3							
IL-4							
IL-5					6		6/24
IL-6				6/24	6		6
IL-7							
IL-9		6		6/24	6/24	6/24	6/24
IL-10		6		6/24			6
IL-12p40				6/24	6	6/24	6/24
IL-12p70				6/24	6	6	6
IL-13							
IL-15				6/24			6/24
IL-17		6		6			
LIF							
LIX				6 ↓			
IP-10				6	6	6	6
KC	6			6/24			6
MCP-1		6		6/24		24	6
M-CSF							6
MIP-1alpha	6			6		6	6
MIP-1beta		6		6/24			6
MIP-2		6		6/24	6/24	6/24	6/24
MIG		6		6/24	6/24	6	6
Rantes		6		6/24	6	6	6
TNF-alpha		6		6/24	6	6	6
VEGF							

The experiment was carried out as described under Materials and Methods and the text. Tumor-bearing mice (5 per group) were treated with QD indomethacin and/or Q3-4D anti-PD-1, starting one day after randomization, and/or once per week Decoy10 starting two days after randomization. Plasma was isolated from tumor-bearing mice (5 per group) 6 and 24 hours after the first complete administration of each single, double or triple combination. Cytokine and chemokine levels were assessed using a 32-plex Luminex panel. Statistical differences for each analyte concentration relative to no treatment were determined by Bartlett test followed by Mann-Whitney U test.

*At either 6 and/or 24 hours after first treatment/Determined by unpaired, non-parametric, Mann-Whitney t-test (p value < 0.05) 5 mice per group in all groups.

**Empty cells represent no significant cytokine/chemokine induction relative to No Treatement.

**Figure 7:**
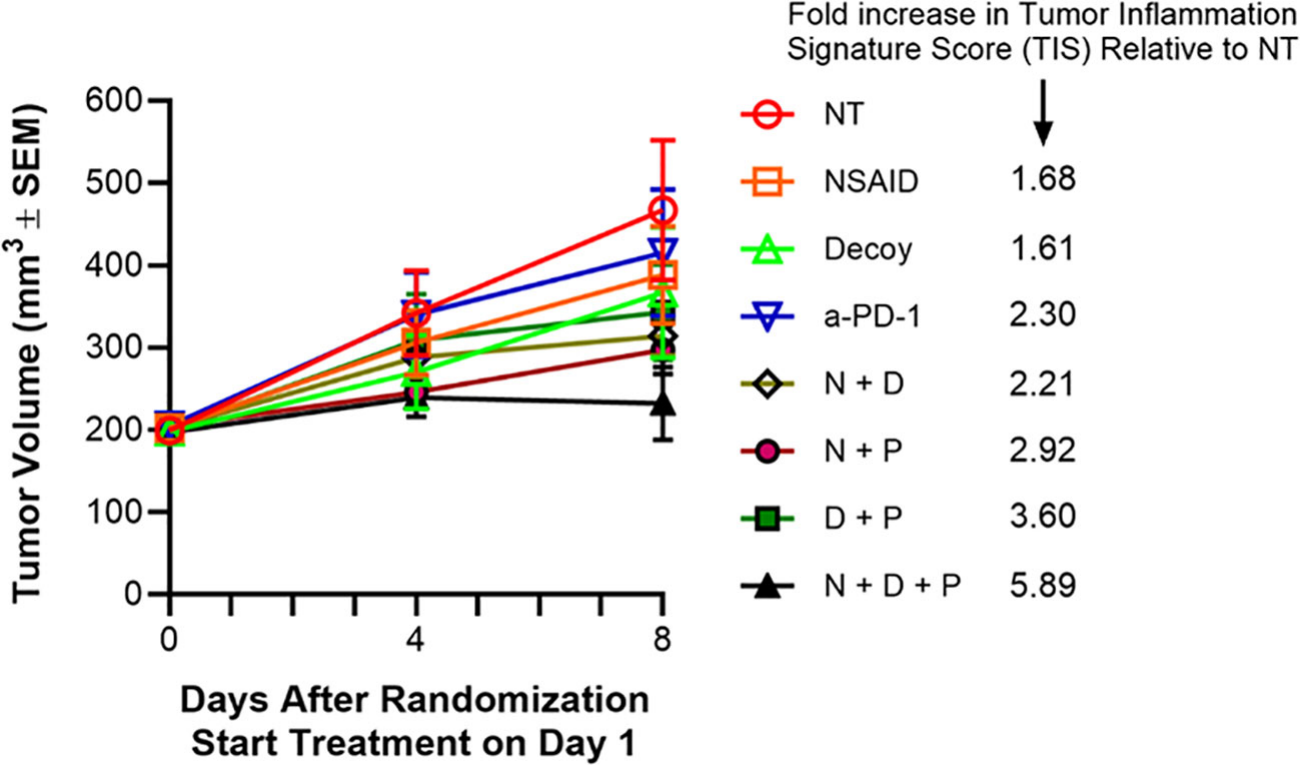
Tumor-eradicating combination therapy increases tumor inflammation signature (TIS) score. The experiment was carried out as described under Materials and Methods and in the text (extension of the experiment described for **Table 5**). Indomethacin (NSAID or N) was administered p.o. in drinking water QD x 7 at 10 µg/mL starting on Day 1. Anti-PD-1 (P) was administered i.p. on days 1 and 4 at 10 mg/kg. Decoy10 (D) was administered i.v. once at 2x10^8^ on Day 2. RNA was isolated from tumors harvested on Day 8 and Tumor Inflammation Signature Score (TIS) analysis was carried out using nanoString technology. The TIS p values for each treatment compared to no treatment were 0.037 NSAID, 0.053 Decoy, 0.001 anti-PD-1, 0.002 NSAID + Decoy, <0.001 NSAID + anti-PD-1, <0.001 Decoy + anti-PD-1, <0.001 NSAID + Decoy + anti-PD-1.

The third satellite group for each original group, treated for one week starting 1 day after randomization, was followed until Day 8, including body weight and tumor measurements (**Figure 7**). Transient average group body weight loss or gain at 4 and 8 days after randomization relative to the day of randomization was -1.63% and 0.97% for the no treatment group, -0.65% and 0.96% for indomethacin, -8.63% and -3.22 for Decoy10, -1.74% and 1.68% for anti-PD-1, -5.05% and -0.24% for indomethacin + Decoy10, -1.29% and 0.82% for indomethacin + anti-PD-1, -7.63% and -2.37% for Decoy10 + anti-PD-1, and -7.98% and -1.52% for the triple combination. There was no increased weight loss for any of the Decoy10 combination treatment groups relative to Decoy10 alone at either Day 4 or Day 8. In addition, there were no other clinical signs of toxicity observed with any mice in any group (checked daily). It is notable that significant increases in cytokine and chemokine expression (in plasma) were observed in the combination groups without any increase in body weight loss or any other clinical signs of toxicity. Tumor growth inhibition after one week of treatment (one dose of Decoy10, two doses of anti-PD-1 and daily indomethacin), relative to no treatment was 17% for indomethacin, 21% for Decoy10, 11% for anti-PD-1, 33% for indomethacin + Decoy10, 36% for indomethacin + anti-PD-1, 26% for Decoy10 + anti-PD-1 and 50% for indomethacin + Decoy10 + anti-PD-1. At termination, after only one week of treatment, 5 out of 6 tumors in the 3-way combination group were smaller in size, compared to the measurements on Day 4 (3 days after initiation of treatment), consistent with the highly efficient and reproducible nature of the combination therapy when treatment is extended. Due to the short duration of treatment, none of the 1-week tumor inhibition results were statistically significant.

The mice in the third satellite groups were sacrificed on Day 8, tumors were harvested, RNA was isolated, and the 48 samples were analyzed using the nanoString PanCancer IO360 panel plus 20 custom genes, and including 20 control genes. NanoString analysis demonstrated statistically significant increases in tumor inflammation signature (TIS) (cold to hot tumor), after only one week of treatment, roughly associated with the degree of tumor growth inhibition at one week and the expected % regressions ultimately expected for the various groups after multiple weeks of treatment (**Figure 7**). An increased TIS is associated with potential for an adaptive immune response (79, 80).

**Figure 8:**
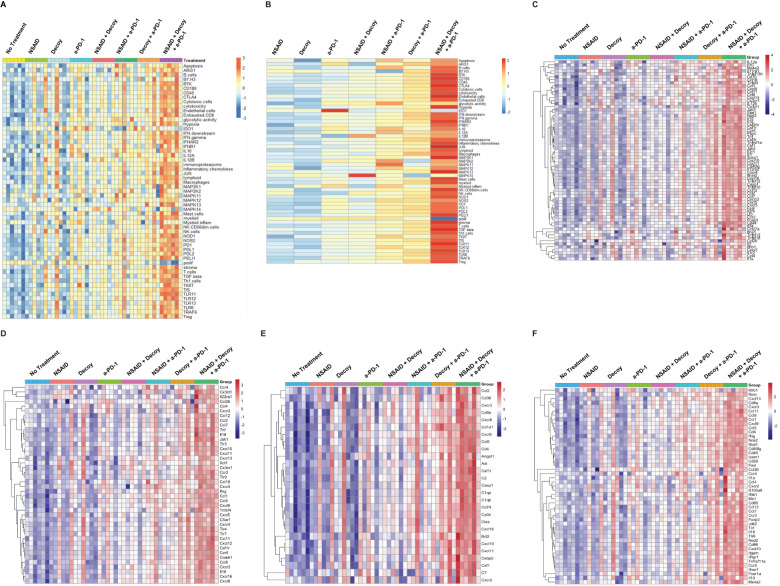
Tumor-eradicating combination therapy is associated with increased innate and adaptive immune pathway gene and cell signature expression in HCC tumors. The experiment was carried out as described under Materials and Methods, in the text, and **Figure 7**. Tumors were harvested after one week of treatment, RNA was isolated and analyzed using nanoString technology. **(A)** Analysis of general immune pathways, cells and genes in individual tumors (6 mice per group). **(B)** Results in 8A averaged within each group. **(C–F)**. Expression of cytokine, chemokine, innate and adaptive pathway-associated genes, respectively, in individual tumors.

Additional nanoString gene expression analysis was carried out evaluating a wide variety of innate and adaptive immune system genes, cells, and pathways. The results were validated based on RNA quality and analysis of house-keeping gene expression. Heatmaps with scaled signature scores were generated representing Log2-based changes in gene expression. Results for general immune-related genes, cells and pathways are shown in **Figure 8A**. Dark blue (-3) is 8-fold below the mean and orange (+3) is 8-fold above the mean. Single agent treatment resulted in broad increases in immune gene/cell/pathway expression in 1 or 2 tumors/mice per group of 6, possibly associated with some tumor growth inhibition. Double agent treatment increased the number of tumors/mice per group exhibiting broad immune gene/cell/pathway activation, possibly associated with increased tumor growth inhibition and some tumor regressions. The triple agent combination was associated with broad immune gene/cell/pathway activation in essentially all tumors/mice, consistent with the high percentage regression and tumor eradication seen in this setting (**Figures 4**–**6**).

When the results in **Figure 8A** were averaged across the six tumors in each treatment group, two results, in particular, stood out (**Figure 8B**). Treatment with single agent anti-PD-1 significantly induced IDO1 (p=0.012), which was not seen in any of the other groups, including combinations with anti-PD-1, suggesting that one of the advantages of combination with either indomethacin or Decoy10 or both may be prevention of induction of this pro-tumor factor (81). It is also clear that one week of treatment with the triple combination was much better, on average, at suppressing proliferation than any of the single or double combinations, being the only group with a statistically significant reduction in proliferation-associated genes (p<0.001). Analysis by nanoString demonstrated significant differences in gene expression for each treatment relative to no treatment. Individual gene expression signatures that exhibited statistically significant changes in each treatment group relative to no treatment are shown in **Supplementary Table S2**. In addition to decreased proliferation, there were a small number of signatures that were only altered (increased) by triple combination treatment (endothelial cells, IFN-beta, JUN, MAP2K1, mast cells, and NK cells).

The individual tumor immune gene activation heatmap patterns in the various groups were the same for cytokine gene/pathway activation (**Figure 8C**), chemokine gene/pathway activation (**Figure 8D**), innate gene/pathway activation (**Figure 8E**) and adaptive gene/pathway activation (**Figure 8F**), all similar to what was observed for general immune-related gene, cell, and pathway signatures (**Figure 8A**).

Two additional combination therapy approaches were explored using low-dose cyclophosphamide (LDC) and rituximab with the syngeneic, s.c., A20 NHL model and the human, s.c., Ramos NHL xenograft model. LDC has been used pre-clinically and in clinical trials to enhance cancer immunotherapy and is thought to function, in part, by reducing levels of T regulatory (Treg) cells (82, 83). It has also been shown to enhance TLR-mediated anti-tumor immune responses (84). LDC was administered i.p. at 20 mg/kg every day (QD) four days per week, one day before, during and one day after QDx2 twice per week Decoy10. Single agent LDC produced statistically significant tumor growth inhibition and an occasional regression in this model. **Supplementary Figure S2A** demonstrates that, when starting treatment with average 212 mm^3^ s.c. tumors, while 3x10^8^ Decoy10 produced no tumor growth inhibition, and LDC produced tumor growth delay without any regressions (Log-rank p<0.001 relative to no treatment), the combination of LDC treatment with Decoy10 produced 6/6 regressions after only two weeks of treatment. The regressions were durable to at least day 125 after tumor cell implant. In addition, the result with combination therapy was statistically significant relative to either single agent treatment (Log-rank p<0.001). Maximum average, transient group body weight loss for the Decoy10 + LDC combination was higher than for other combinations (12-14%). Despite observation of similar, significant, transient body weight loss in Decoy10 + LDC combination groups in multiple studies, no deaths or other clinical signs of toxicity were observed except transient ruffled fur. Surprisingly, higher doses of QDx2 Decoy10 were tolerated without lethality in combination with LDC compared to Decoy10 alone, despite higher transient weight loss. High percentage regression of established NHL tumors was observed with Decoy10 or Decoy20 + LDC in three independent experiments.

Rechallenge of the 6 tumor regressed mice from **Supplementary Figure S2A** with fresh A20 cells on the opposite flank from the first tumor challenge produced 5/6 tumor-free mice, while naïve mice challenged with the same tumor cells on the same day exhibited full tumor growth (**Supplementary Figure S2B**). High percentage immunological memory after A20 tumor rechallenge was observed with mice cured by Decoy10 or Decoy20 + LDC in 2 independent experiments.

An alternative and complementary mechanism of action approach, relative to the one used in **Figures 7**, **8** with HCC tumors, was used with the A20 NHL model to determine if tumor eradication by Decoy10 + LDC was associated with both innate and adaptive immune pathway activation. Satellite groups of mice were pretreated with neutralizing antibodies in order to deplete NK, or CD4^+^ and/or CD8^+^ T cells prior to initiation of Decoy10 + LDC treatment (**Supplementary Figure S3**). Pre-depletion of 98% of NK cells resulted in 3/6 complete regressions, but with only 1/6 durable; 100% pre-depletion of CD4+ T cells resulted in 1/6 transient or durable regressions; 92% pre-depletion of CD8+ T cells resulted in 2/6 tumor regressions, with only 1/6 durable; and 100% pre-depletion of both CD4+ and CD8+ T cells prevented all tumor regressions/responses. These results complement the gene expression results obtained with the H22 HCC model, demonstrating that efficient tumor eradication by Decoy combination therapy involves activation of both innate and adaptive immune pathways. Comparison of the immune cell depletion curves to the no treatment control in the same experiment (**Supplementary Figure S2A**) suggests that there was some tumor growth delay even in the 93-100% absence of NK, CD4^+^ T and/or CD8^+^ T cells, suggesting that additional mechanisms contribute to tumor growth inhibition.

The experiment in **Supplementary Figure S2** was repeated in order to determine if mice readily develop resistance to sub-optimal Decoy10 + LDC treatment, and if large subcutaneous tumors can be regressed by Decoy10 combination therapy. Eight mice with established tumors were treated with a suboptimal or optimal dose/regimen of Decoy10 + standard LDC for only one week. All eight of the tumors partially or fully regressed, but then started to regrow, producing tumors that ranged in size from 122 to 1,782 mm3 on Day 49 (**Supplementary Figure S4**). Re-treatment with the optimal two-week Decoy10 + LDC doses and regimen was initiated on Day 50. All but one of the tumors continued to grow for 4-7 days after treatment was re-initiated and then all of the tumors started to regress, with 5 of 8 producing full regressions, including tumors that reached 141, 281, 485, 780 and 2,568 mm^3^ in volume during retreatment. Positive control groups receiving two different saturating doses of Decoy10 + standard LDC for two weeks exhibited 8/8 full regressions, and all 8 mice fully rejected a second tumor challenge, demonstrating reproducible immunological memory (data not shown).

If single agent anti-tumor activity and/or combination-mediated tumor eradication with Decoy bacteria involve activation of innate immune pathways, then it was hypothesized that Decoy bacteria might produce anti-tumor activity in a human tumor xenograft model, where only an innate immune system is present. This was tested with an established, human Ramos NHL xenograft model in SCID mice, which lack B and T cells (5 mice per group). Single agent Decoy10 produced modest, but statistically significant anti-tumor activity (Log-rank p=0.013), without any regressions. Single agent Rituximab or LDC also inhibited tumor growth (Log-ranks p=0.002), but did not produce any regressions. The combination of Decoy10 + LDC produced 3/5 complete regressions and 2/5 partial regressions, with all tumors regrowing after Days 33-57. The triple combination of Decoy10 + LDC + rituximab produced 5/5 complete regressions, which were durable until Days 85 to 96, with one durable until termination at Day 113. Survival with both double combinations was significantly enhanced relative to each of the single agents (Log ranks p<0.005), and survival in the triple combination was significantly enhanced relative to the two tested double combinations (Log-ranks p<0.05). The maximum transient average group body weight loss was 7.9% (LDC), 5.6% (Decoy10), 15.1% (Decoy10 + LDC), 1.1% (Rituximab), 12% (Decoy10 + Rituximab), and 12% (Decoy10 + LDC + Rituximab). There were no drug-related deaths or requirement for dose holidays. High percentage regression of established Ramos tumors by the triple combination was observed in two independent experiments.

The five triple combination treated mice with 5/5 complete regressions at Day 74 were rechallenged with fresh Ramos tumor cells on the opposite flank from the first tumor challenge (no new treatment). Naïve mice received a first tumor cell challenge on the same day. The naïve mice had to be sacrificed within 25-32 days due to tumor volume >3,000 mm3. At Day 39 post rechallenge, three out of five of the rechallenge sites, on mice which had previously experienced tumor regression, were tumor free, demonstrating partial innate only immunological memory or trained immunity. In addition to demonstrating innate immunological memory, these results demonstrate that tumor growth inhibition and regression by Decoy bacteria and combinations is not limited to murine tumors but can also be achieved with human tumors in an innate only setting.

## 4 Discussion

The hypothesis addressed in this communication is that improvements in immunotherapy of advanced or metastatic tumors might be facilitated by safe, systemic administration of a package of diverse immune agonists, under conditions where continuous or long-term exposure is avoided. This hypothesis takes advantage of known properties of Gram-negative bacteria. Namely, that these bacteria contain a wide variety of TLR and other immune receptor agonists, are cleared very rapidly by the liver and spleen after systemic administration, and have been reported to have anti-tumor activity with i.t. or s.c. administration in the form of Coley’s Toxins. Coley reported that his approach appeared to work best when administered systemically but produced toxicity that was difficult to control (21). Since both efficacy and dose-limiting toxicity of systemically administered Gram-negative bacteria are likely to involve the activity of LPS-endotoxin, and since it is probably present in huge excess, as the major component of the outer membrane, it made sense to significantly reduce, but not completely eliminate its activity.

The method developed, involving ~96% reduction of TLR4 agonist activity with PMB, followed by killing of non-pathogenic, Gram-negative bacteria with GA, produced product candidates with several unique properties, including stabilization, which might facilitate passive targeting or delivery of intact bacteria to the liver and spleen, where they should be rapidly processed by immune cells such as Kupffer macrophages (85). This is expected to produce broad, but transient local and systemic immune activation, at least in part, via induction of cytokine and chemokine secretion (passively targeted Pulse-Prime hypothesis). As predicted, Decoy bacteria exhibited reduced toxicity *in vivo*, but surprisingly no reduction in ability to induce PBMC secretion of 7 out of 8 major cytokines and chemokines assessed, and apparent increases in secretion of 6 out of 8. The mechanistic basis for this observation has not been determined, but it may involve delayed degradation and extended immune receptor activation by the bacteria due to GA-mediated stabilization. Regardless of the mechanism, the results suggest that the manufacturing process may partially uncouple toxicity from immune activation.

Single agent anti-tumor activity was observed with Decoy10 and Decoy20 with three different indications (colorectal, pancreatic, and hepatocellular carcinoma) using three different implantation models (s.c., orthotopic, and metastatic), with a therapeutic index of approximately 10-fold. Durable regression of relatively large, established s.c. tumors was observed with Decoy10 or Decoy20 in combination with low-dose, oral indomethacin, anti-PD-1, or indomethacin + anti-PD-1 in the s.c. syngeneic hepatocellular carcinoma model, and with low-dose cyclophosphamide or low-dose cyclophosphamide + rituximab in syngeneic NHL and human xenograft NHL models, respectively. Combination-mediated durable regressions appeared to be the result of synergistic interactions in some settings, based on no or very infrequent regressions with the individual combination components, and statistically significant Log-rank p values with the combinations relative to single components in the double combinations, and in some instances relative to the double combinations in the triple combination. There was no significant increase in toxicity when Decoy10 or Decoy 20 were combined with indomethacin and/or anti-PD-1, and this conclusion is supported by the finding of a ≥33-fold therapeutic index for 80-100% eradication of established HCC tumors in the triple combination setting. Combination of Decoy10 with LDC produced increased transient weight loss, relative to Decoy10 alone but surprisingly, Decoy10 was tolerated at higher doses in combination with LDC than as a single agent in this model.

Combinations that produced tumor regressions in the HCC model also produced synergistic and mostly transient induction of plasma cytokines and chemokines, and this may be important for or associated with anti-tumor activity. The same cytokines/chemokines can stimulate innate or adaptive anti-tumor immune responses, contribute to pro-tumorigenic immune suppression in the tumor microenvironment (TME), enhance tumor angiogenesis, or produce systemic toxicity, depending on the time, place, and/or duration of expression. For example, several cytokines and chemokines generally considered to be predominantly toxic or immunosuppressive (IL-6, IL-8 and IL-10) have been shown to stimulate anti-tumor immune responses in certain settings (86–88). The triple combination of Decoy + NSAID + anti-PD-1, producing 80-100% durable tumor regressions with 4-6 weeks of treatment, induced plasma expression of 23 cytokines/chemokines, with at least 17/23 being only transiently induced (not determined for the analytes observed at both 6 and 24 hours). Toxic effects of cytokines/chemokines, such as cytokine-release syndromes (CRS), are typically associated with continuous systemic exposure for at least several days, and this may also be true for immunosuppressive and pro-angiogenic consequences in the TME (89–92). The finding that single agent Decoy bacteria only induced mouse plasma cytokine and chemokine expression for less than 24 hours, suggests that single agent Decoy may not induce CRS in humans. The results presented in this communication suggest that pulsed, transient expression of a broad or diverse range of cytokines and chemokines may participate in facilitation of anti-tumor immune responses without induction of unacceptable systemic toxicity.

High percentage (80-100%) tumor rechallenge rejection or immunological memory was observed in multiple syngeneic combination models, including with either Decoy10 or Decoy20. Tumor regressions in multiple syngeneic tumor models was reported previously using i.p.-administered single or double monospecific TLR agonist combinations [CpG or CpG + poly(I:C)] with cytotoxic doses of cyclophosphamide (200 mg/kg), but this approach only produced 10-30% immunological memory on rechallenge (51).

Delivery of multiple, different TLR agonists, including LPS, was predicted to activate both innate and adaptive immune pathways. This was confirmed by gene expression analysis in established tumors after administration of only one dose of Decoy10. However, only partial innate and adaptive activation was observed in most mice, with close to full activation in only one of six mice. A similar pattern was observed with single agent indomethacin or anti-PD-1. This may partially explain why the single agents produced tumor growth delay, but no tumor regressions. Innate and adaptive pathway activation increased in most of the mice with the double combinations, and this was associated with 2/6 durable regressions for each of these regimens when extended beyond one week of treatment. Nearly saturating activation of both innate and adaptive pathways, with respect to the nanoString heatmaps, was observed with all mice treated for one week with the triple combination, and this pattern was associated with a high percentage of durable regressions upon treatment extension. Two doses of anti-PD-1 produced stronger general immune pathway activation than either single agent indomethacin or Decoy10, but also produced statistically significant induction of IDO1, which was not seen in the double combinations or triple combination. The synergistic nature of the triple combination is also supported by the averaged results, including the statistically significant reduction in proliferation-related genes, and induction of *IFNB1* and the NK cell gene signature.

The double and triple combination treatments induced genes associated with both anti-tumor and immune-suppressive activity, at least in the first week of treatment. This global activation was ultimately associated with significant anti-tumor activity and induction of immunological memory. Gene expression analysis after more than one week of treatment will be required to determine if induction of genes generally considered to be immune-suppressive is preferentially lost with repeat treatment. The predicted pulsatile nature of Decoy exposure may play a role in biasing the ultimate balance in favor of anti-tumor immunity. Regardless, the results demonstrate that induction of immune-suppressive genes, at least initially, does not necessarily prevent anti-tumor immunity.

A role for activation of both innate and adaptive immune pathways in combination-mediated tumor eradication by Decoy bacteria was demonstrated by gene expression analysis in the HCC model, by immune cell depletion studies with the syngeneic NHL model, and with regression of tumors in the human tumor xenograft NHL model. Innate immunological memory, also referred to as trained immunity, was observed with the NHL xenograft model. This is an established phenomenon, including with TLR activation, in infectious disease and anti-tumor settings (66, 93–98). We have obtained additional evidence for activation of specific innate and adaptive immune cell types by Decoy10 using human PBMCs *in vitro*, via demonstration of induction of activation, polarization, or maturation markers in NK, NKT, dendritic, CD4+ T and CD8+ T cells (99).

IND-enabling toxicology studies with Decoy20 have produced support for the passive targeting hypothesis, based on histopathological observation of non-adverse immune activation in the liver and spleen of rabbits, without similar activation in other organs (Newman M.J., unpublished data). These results also suggest potential to target primary tumors or metastatic disease in the liver. Decoy20 is currently being evaluated in a US Phase 1 clinical trial in patients with advanced solid tumors (NCT05651022). Preliminary clinical results have provided support for the Pulse-Prime hypothesis, as pharmacokinetic analysis with tolerated single doses of Decoy20 demonstrated disappearance of Decoy20 from blood within 30 to 120 minutes after the end of a one hour infusion, associated with transient induction of over 50 plasma cytokines and chemokines, without report of cytokine release syndrome (100, 101). The breadth of transient plasma cytokine/chemokine induction observed after a single dose of Decoy20 in our Phase 1 trial is similar to the plasma cytokine/chemokine profiles observed for the tumor regression-associated double or triple combination therapy profiles in **Table 5** of this communication.

In summary, this work provides a novel approach for pulsed, systemic administration of a package of innate and adaptive immune cell receptor agonists, which may avoid some forms of toxicity associated with therapeutics that depend on continuous exposure for activity. Observation of regressions, tumor eradications and immunological memory with Decoy bacteria in combination with four different approved drug classes also suggests broad potential for this approach in a variety of different oncology settings.

## Data Availability

The raw data supporting the conclusions of this article will be made available by the authors, without undue reservation.
