# Supplementary material for: Invention and characterization of a systemically administered, attenuated and killed bacteria-based multiple immune receptor agonist for anti-tumor immunotherapy
Source: Front Immunol. 2024 Nov 7;15:1462221. doi: 10.3389/fimmu.2024.1462221 (PMC11599860; doi:10.3389/fimmu.2024.1462221)
Supplement: Supplementary file 1 [file DataSheet1.pdf]

Supplementary Material

SUPPLEMENTARY TABLE 1. Twenty custom genes added to the NanoScreen nCounter PanCancer Mouse IO360 Panel.

Number	Customer Identifier	Accession	Position
1	*BTK*	NM_013482.2	2256-2355
2	*Cd180*	NM_008533.2	861-960
3	*Ifnar2*	NM_001110498.1	726-825
4	*Ifnb1*	NM_010510.1	336-435
5	*IL12a*	NM_008351.1	356-455
6	*IL12b*	NM_001303244.1	415-514
7	*Jun*	NM_010591.2	2213-2312
8	*Map2k1*	NM_008927.3	1696-1795
9	*Map2k2*	NM_023138.4	1441-1540
10	*Mapk11*	NM_011161.5	2108-2207
11	*Mapk12*	NM_013871.3	1587-1686
12	*Mapk13*	NM_011950.1	396-495
13	*Mapk14*	NM_011951.2	1421-1520
14	*Nod1*	NM_172729.2	1447-1546
15	*Peli1*	NM_023324.2	591-690
16	*Tlr11*	NM_205819.2	1681-1780
17	*Tlr12*	NM_205823.2	381-480
18	*Tlr13*	NM_205820.1	2786-2885
19	*Tlr6*	NM_011604.3	476-575
20	*Traf6*	NM_009424.2	981-1080

SUPPLEMENTARY TABLE 2. Treatment-induced gene expression changes in tumors isolated as described in Figure 7 (nanoString analysis). Data was derived from six individual tumors for each treatment group and signatures are only listed if still significant compared to No Treatment after Benjamini and Hochberg False Discovery Rate (FDR) adjustment. Signatures highlighted in yellow were only observed in one treatment group.

NSAID	Decoy10	Anti-PD-1	Decoy10 + Anti-PD-1	NSAID + Decoy10	NSAID + Anti-PD-1	NSAID + Decoy10 + Anti-PD-1
**Number of HCC Tumor Regressions Per Group With 4-6 Weeks Treatement in Separate Experiments**						
**0 / 6**	**0 / 6**	**0 / 6**	**2 / 6**	**2 / 6**	**2 / 6**	**5 to 6 / 6**
**Statistically Significant Gene Expression Changes in Tumors After FDR Adjustment (After One Week of Treatment - Relative to No Treatment)**						
Apoptosis	*IL-12B*	*Apoptosis*	*Apoptosis*	*Apoptosis*	*Apoptosis*	*Apoptosis*
B.cells		*ARG1*	*ARG1*	*BTK*	*ARG1*	*ARG1*
Exhausted.CD8		*ARG1*	*B.cells*	*CD45*	*B.cells*	*B.cells*
Hypoxia		*BTK*	*BTK*	*cytotoxic.cells*	*BTK*	*BTK*
IL12B		*cytotoxic.cells*	*CD180*	*cytotoxicity*	*CD45*	*CD180*
NK.CD56dim.cells		*cytotoxicity*	*CD45*	*Exhausted.CD8*	*cytotoxic.cells*	*CD45*
		Exhausted.CD8	*CTLA4*	*glycolytic.activity*	*cytotoxicity*	*CTLA4*
		glycolytic.activity	*cytotoxic.cells*	*Hypoxia*	*Exhausted.CD8*	*cytotoxic.cells*
		IDO1	*cytotoxicity*	*IFN.gamma*	*glycolytic.activity*	*cytotoxicity*
		IFN.gamma	*Exhausted.CD8*	*IL12B*	*Hypoxia*	*Endothelial.cells*
		IL10	*glycolytic.activity*	*immunoproteasome*	*IFN.downstream*	*Exhausted.CD8*
		immunoproteasome	*Hypoxia*	*inflammatory.cytokines*	*IFN.gamma*	*glycolytic.activity*
		inflammatory.cytokines	*IDO1*	*lymphoid*	*IL10*	*Hypoxia*
		lymphoid	*IFN.downstream*	*NK.CD56.dim.cells*	*IL12A*	*IFN.downstream*
		NOD1	*IFN.gamma*	*NOD1*	*IL12B*	*IFN.gamma*
		NOS2	*IL10*	*PD1*	*immunoproteasome*	*IFNB1*
		PD1	*IL12A*	*PDL2*	*INFAR2*	*IL10*
		PDL1	*IL12B*	*PELI1*	*inflammatory.cytokines*	*IL12A*
		PDL2	*immunoproteasome*	*T.cells*	*lymphoid*	*IL12B*
		T.cells	*INFAR2*	*Th1.cells*	*MAP2K2*	*immunoproteasome*
		Th1.cells	*inflammatory.cytokines*	*TIGIT*	*MAPK12*	*INFAR2*
		TIGIT	*lymphoid*	*TIS*	*myeloid*	*inflammatory.cytokines*
		TIS	*macrophages*	*TLR12*	*NK.CD56.dim.cells*	*JUN*
		TLR12	*MAPK13*	*TRAF6*	*NOD1*	*lymphoid*
		TRAF6	*Mast.cells*	*TRL11*	*NOS2*	*macrophages*
		TRL11	*myeloid*		*PD1*	*MAP2K1*
			NK.CD56.dim.cells		*PDL1*	*MAP2K2*
			NOD1		*PDL2*	*MAPK12*
			NOS2		*PELI1*	*MAPK13*
			PD1		*T.cells*	*Mast.cells*
			PDL1		*Th1.cells*	*myeloid*
			PDL2		*TIGIT*	*NK.CD56.dim.cells*
			PELI1		*TIS*	*NK.cells*
			Stroma		*TLR6*	*NOD1*
			T.cells		*TRL11*	*NOS2*
			TGF.beta		*TLR12*	*PD1*
			Th1.cells		*TRAF6*	*PDL1*
			TIGIT			*PDL2*
			TIS			*PELI1*
			TLR6			*↓proliferation*
			TRL11			*Stroma*
			TLR12			*T.cells*
			TLR13			*TGF.beta*
			TRAF6			*Th1.cells*
			TRAFT6			*TIGIT*
			Treg			*TIS*
						Treg
						TRL11
						TLR12
						TLR13
						TLR6
						TRAF6

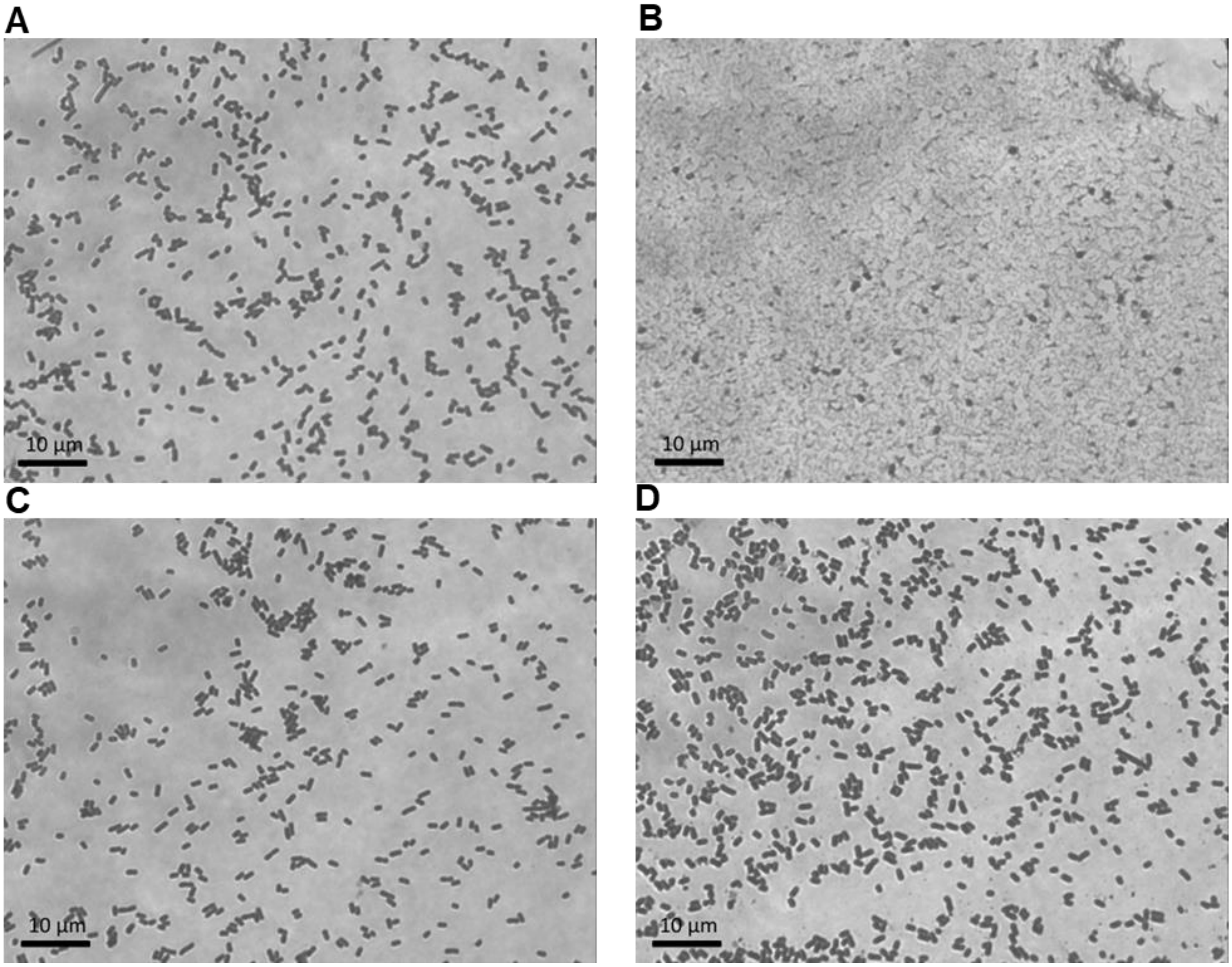

SUPPLEMENTARY FIGURE 1. Light microscope grey-scale images of crystal violet-stained ATCC 13070 bacteria and Decoy10 demonstrating enhanced stability of Decoy10. The bacteria were grown, treated to produce Decoy10, and sonication and processing were carried out as described under Materials and Methods. (A) Untreated ATCC 13070 bacteria. (B) Untreated ATCC 13070 bacteria sonicated for 5 minutes. (C) Decoy10 bacteria. (D) Decoy10 bacteria sonicated for 10 minutes (all images at ~1,000-fold magnification).

A

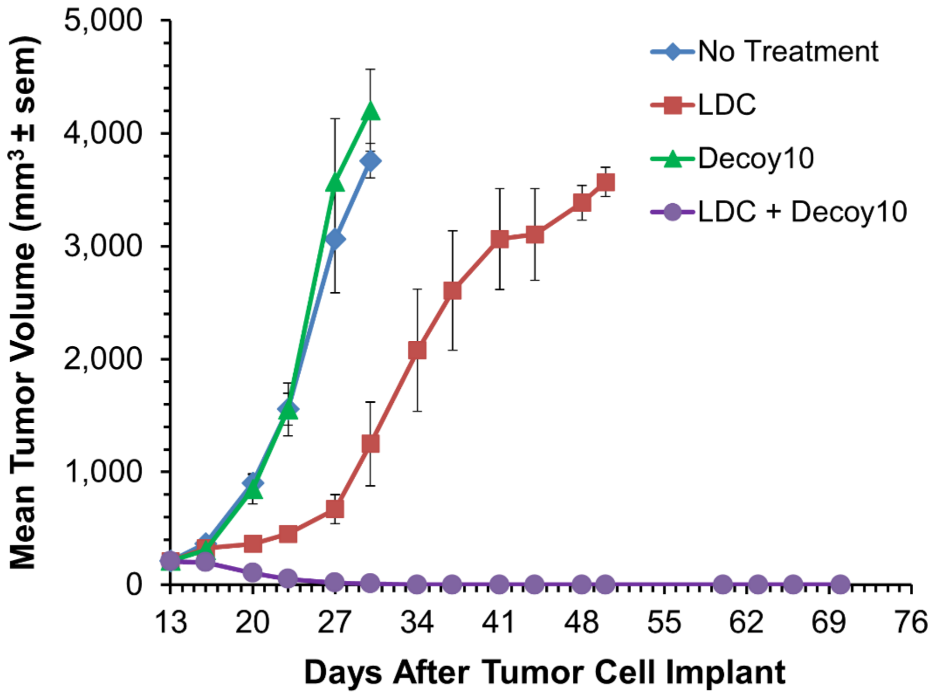

B

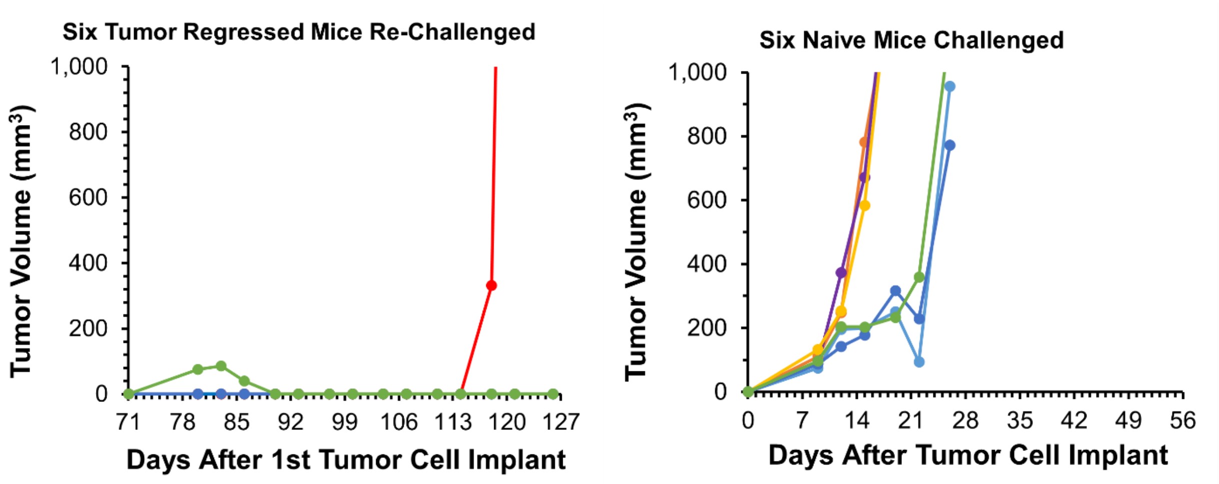

SUPPLEMENTARY FIGURE 2. Decoy10 synergizes with low-dose cyclophosphamide (LDC) to regress established s.c. A20 NHL. The experiment was carried out as described under Materials and Methods. Tumor implantation was carried out with $5x10^5$ cells in PBS. Randomization to 6 mice per group was carried out and treatment was initiated 13 days later when the average tumor volume was 212 mm^3. LDC was administered i.p. at 20 mg/kg QDx4 (four days in a row) each week for two weeks starting on Day 13. Decoy10 was administered i.v. at $3x10^8$ QDx2 each week for two weeks starting on Day 14 (during second and third day of LDC treatment). **(A)** Decoy10 did not produce single agent activity in this model and LDC produced a statistically significant delay of tumor growth compared to no treatment (p=0.001 by Log-rank test), without producing regressions. The combination produced 6/6 full regressions (CR) and was statistically significant relative to LDC

alone (Log-rank p=0.001). Maximum transient, average group body weight loss during each of the two weeks of treatment, relative to the day of randomization, was 0.8% and 0.2% for LDC alone, 6.8% and 3.2% for Decoy10, and 12% and 13.6% for Decoy10 + LDC. **(B)** The 6 tumor-regressed mice treated with LDC + Decoy10 were rechallenged on Day 70 with fresh A20 tumor cells on the opposite flank relative to the first tumor challenge. Naïve mice were challenged with the same cells on the same day. There was no further treatment. All first and 5/6 second tumor challenge sites in the tumor regressed mice were tumor-free at termination on Day 125. Full tumor take was recorded in naïve mice that received the same tumor cells on the same day as the re-challenge.

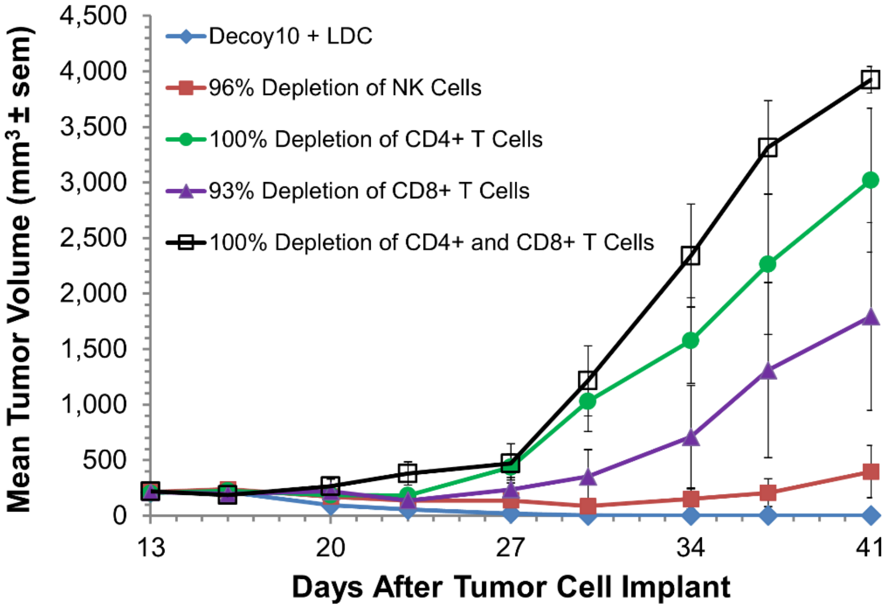

SUPPLEMENTARY FIGURE 3. Efficient synergistic tumor regression induced by LDC and Decoy10 in the A20 NHL model involves innate and adaptive immune pathways. The experiment was carried out as described under Materials and Methods, the text, and Supplementary Figure 2. The experiment described under Supplementary Figure 2 was expanded to include groups with pre-depletion of NK cells, CD4+ and/or CD8+ T cells before and during LDC + Decoy10 treatment. Depletion efficiency was determined with satellite groups at the start of LDC + Decoy10 treatment (same no treatment group). Durable regressions were determined at study termination on Day 70. All of the mice in this portion of the experiment, except 6 of 12 LDC + Decoy10 positive control mice, received daily indomethacin treatment in addition to LDC + Decoy10. This addition was not required for and did not negatively impact initial tumor regression in the control group.

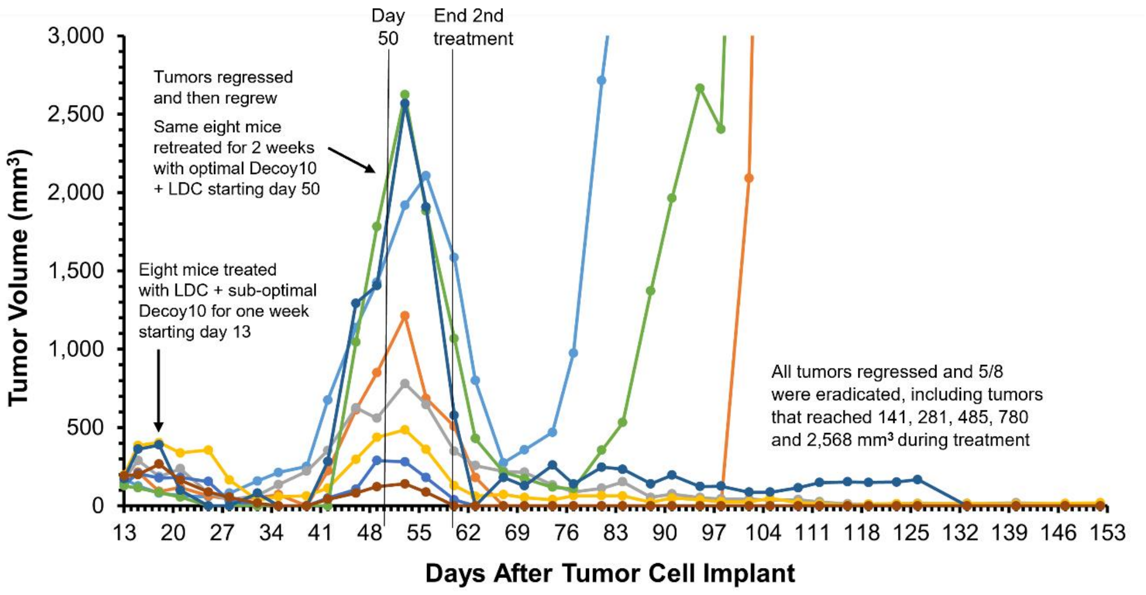

SUPPLEMENTARY FIGURE 4. Optimal LDC + Decoy10 can regress large s.c. A20 NHL tumors after sub-optimal treatment and relapse. The experiment was carried out as described under Materials and Methods, the text and Supplementary Figure 2. Randomization was carried out and suboptimal treatment was initiated with 8 mice with an average tumor volume of 158 mm^3 13 days after tumor cell implantation. Mice were initially treated with 20 mg/kg LDC QDx4 (standard regimen) + $3x10^7$ to $1x10^9$ QDx2, QDx3 or QDx4 Decoy10 for one week (Days 13-19). Treatment was withheld from Day 20 to 49 and then re-initiated on Days 50/51 with 20 mg/kg LDC QDx4 and $3x10^8$ Decoy10 QDx2 for two weeks. Initial partial or full regression followed by relapse was seen with all sub-optimally treated tumors. Optimal treatment started on Day 50 produced 5/8 full regressions at termination on Day 152.

A

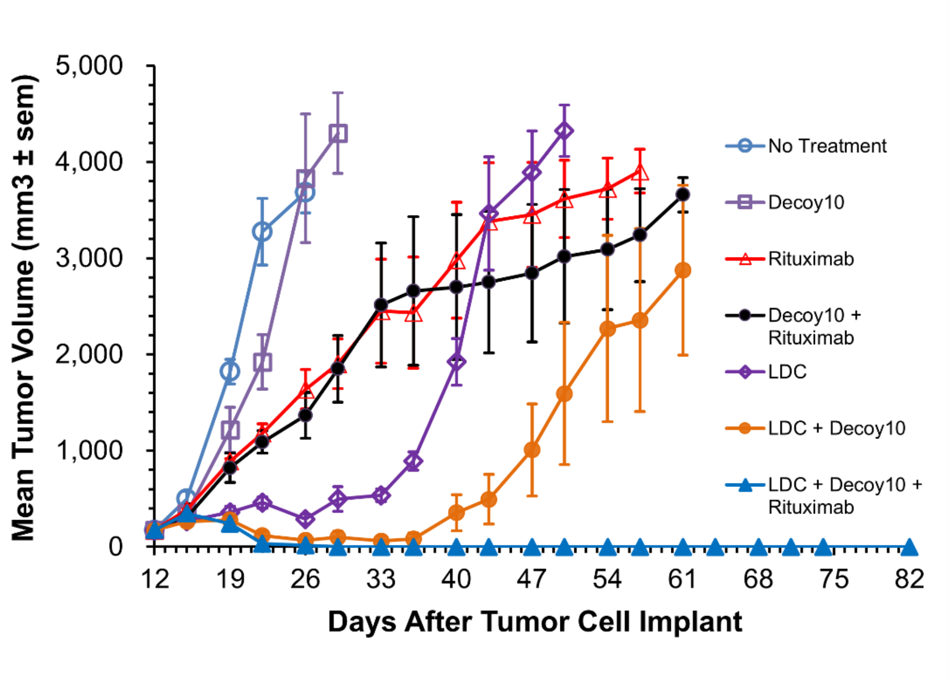

B

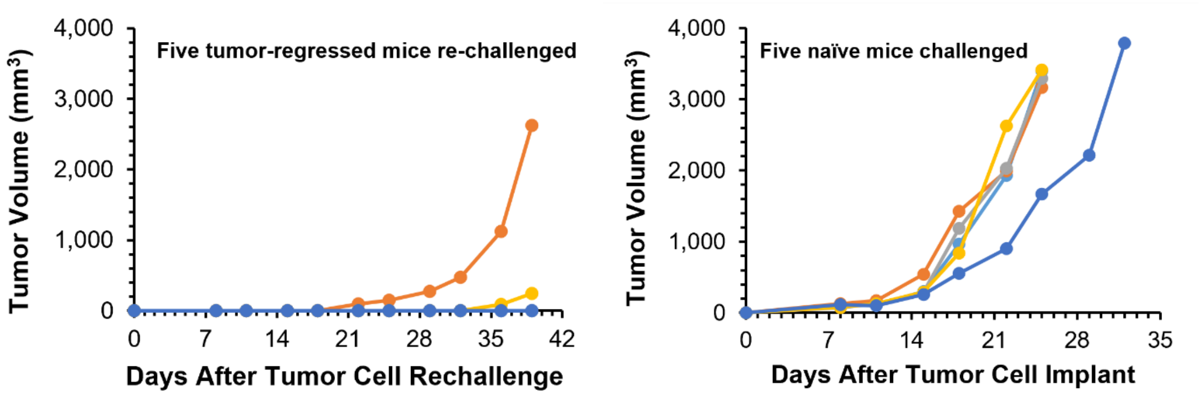

SUPPLEMENTARY FIGURE 5. Decoy10 and LDC synergize with a targeted antibody to regress established s.c. human Ramos NHL xenografts, with induction of partial innate immunological memory. The experiment was carried out as described under Materials and Methods and in the text. **(A)** Tumor implantation was carried out with $1x10^7$ Ramos cells in PBS/Matrigel (1/1). Randomization to 5 mice per group was carried out and treatment was initiated on Day 12 when the average tumor volume was 174 mm^3. LDC was administered i.p. at 20 mg/kg QDx4 each week for three weeks starting on Day 12. Rituximab was administered i.p. at 100 µg/mouse Q3-4 days each week for three weeks starting on Day 12. Decoy10 was administered i.v. at $2x10^8$ QDx2 each week for three weeks starting on Day 13. LDC + rituximab was not tested in this experiment but did not produce any durable regressions in a separate experiment, where rituximab was tested at 200 µg/mouse once per week (data not shown). **(B)** Five triple combination-treated mice with complete regressions were rechallenged on Day 74 with fresh Ramos tumor cells on the opposite flank from the first tumor challenge. Three of five rechallenge sites remained tumor-free up to termination on

Day 113. The same tumor cells were implanted in 5 naïve mice on Day 74, producing >3,000 mm^3 tumors.
